# Hidden electronic rule in the “cluster-plus-glue-atom” model

**DOI:** 10.1038/srep33672

**Published:** 2016-09-19

**Authors:** Jinglian Du, Chuang Dong, Roderick Melnik, Yoshiyuki Kawazoe, Bin Wen

**Affiliations:** 1 State Key Laboratory of Metastable Materials Science and Technology, Yanshan University, Qinhuangdao 066004, China; 2 Key Laboratory of Materials Modification [Dalian University Technology], Ministry of Education, Dalian 116024, China; 3 The MS2Discovery Interdisciplinary Research Institute, Wilfrid Laurier University, 75 University Ave. West, Waterloo, Ontario N2L 3C5, Canada; 4 BCAM-Basque Center for Applied Mathematics, E48009 Bilbao, Spain; 5 New Industry Creation Hatchery Center, Tohoku University, 6-6-4 Aramaki-aza-Aoba, Aoba-ku, Sendai 980-8579, Japan; 6 Institute of Thermophysics, Siberian Branch of the Russian Academy of Sciences, 1, Lavyrentyev Avenue, Novosibirsk 630090, Russia

## Abstract

Electrons and their interactions are intrinsic factors to affect the structure and properties of materials. Based on the “cluster-cluster-plus-glue-atom” model, an electron counting rule for complex metallic alloys (CMAs) has been revealed in this work (i. e. the CPGAMEC rule). Our results on the cluster structure and electron concentration of CMAs with apparent cluster features, indicate that the valence electrons’ number per unit cluster formula for these CMAs are specific constants of eight-multiples and twelve-multiples. It is thus termed as specific electrons cluster formula. This CPGAMEC rule has been demonstrated as a useful guidance to direct the design of CMAs with desired properties, while its practical applications and underlying mechanism have been illustrated on the basis of CMAs’ cluster structural features. Our investigation provides an aggregate picture with intriguing electronic rule and atomic structural features of CMAs.

Composition-structure-properties correlations are important topics with great significance in materials science research fields^1^,^2^,^3^,^4^,^5^. Crystallographic method describes simple crystalline materials’ structure by means of “atomic positions plus space lattice”, and usually knowledge of a few atoms within the unitcell is sufficient to deduce their partial properties^6^,^7^. For complex metallic alloys (CMAs) like some intermetallics, quasicrystals and amorphous alloys, the problem becomes complicated because their structural information is often submerged in a long list of atomic coordinates. In this case, the structural characteristics of CMAs cannot be reflected through the crystallographic method, not to mention their structure-related properties^8^,^9^,^10^. Since the atomic clusters are advocated as primary units to represent materials’ structural features, to solve the above problem, various cluster-based models have been developed during the past decades^11^,^12^,^13^,^14^,^15^,^16^,^17^. Among these cluster-based models, Dong’s “cluster-plus-glue-atom” model^15^ can be used to describe the atomic structure of nearly all materials. Denoted by a uniform cluster formula of [cluster](glue atoms)_x_^18^,^19^,^20^,^21^, this cluster-plus-glue-atom model regards the atomic structure of any materials, no matter whether crystalline or non-crystalline, to be composed of the clusters part and the glue atoms part^22^,^23^,^24^,^25^,^26^,^27^,^28^. Accordingly, all atoms in a given structure belong to three kinds of the central atoms, the shell atoms and the glue atoms^29^,^30^, as the red spheres, the blue spheres and the green spheres shown in Supplementary Figure S1, respectively. In this context, the cluster-plus-glue-atom model contains materials’ basic composition information and structure information in its cluster formula, thus it lays the foundation to uncover the connections among composition, structure and properties of materials, especially for those CMAs with complicated atomic configuration.

Electrons and their interactions are believed as the most intrinsic factors to dominate the structure and properties of materials^31^,^32^,^33^,^34^,^35^. When different atoms gather into molecules, there are electrons transferring and these electrons share or overlap in the bond-making process. Consequently, different materials behave different properties, and for a long time, electron factors have attracted considerable attentions to investigate the composition-structure- properties correlations of materials^36^,^37^. For example, the exclusive principle proposed by Pauli at the beginning of last century, has been successfully used to extend the formal classification of valence electrons by four quantum numbers^38^,^39^,^40^,^41^. Afterwards, the valence bond theory and molecular orbital theory have been developed successively to explore the structure and properties of materials^42^,^43^. One of the most important corollaries of these electronic theories, is that the stable electronic configuration of common covalent compounds and ionic compounds follow the octet rule^44^,^45^,^46^. As for alloy phases, Hume-Rothery points out that electron concentration plays an important role in stabilizing the structure of electron compounds, which is known as the Hume-Rothery rule^47^,^48^,^49^. Actually, many structural distinctions of metals and alloys can be discussed directly from their electron concentration differences^32^,^47^,^50^. Furthermore, the structure and structure related properties of CMAs originate from the local atomic bonding inside and between the cluster structural units via electronic interactions^51^,^52^,^53^. Therefore, it becomes necessary to investigate CMAs’ composition-structure-property correlations from the electron perspective.

Depending on the cluster-plus-glue-atom model, the cluster formula of CMAs is equivalent to the molecular formula of covalent compounds and ionic compounds. Meanwhile, it is known that a majority of covalent compounds and ionic compounds follow the octet rule. Accordingly, we speculate that CMAs should follow analogous electronic rule as well. In this work, an electron counting rule hidden in the cluster formula for CMAs is revealed. Our analysis on the cluster structure and electron concentration for typical kinds of CMAs^20^,^22^,^24^,^26^,^54^ (including intermetallic compounds, quasicrystals and metallic glasses), indicates that the valence electrons’ number per unit cluster formula for these CMAs are close to specific constants of eight-multiples and twelve-multiples. It is thus termed as CMAs’ specific electrons cluster formula. This electron counting rule has been demonstrated as a useful guidance to direct the design of CMAs with desired properties, and its practical application has been illustrated accordingly. Furthermore, an underlying mechanism behind this electron counting rule is presented on the basis of CMAs’ cluster structural features. The present work will help people to better understand the composition-structure-properties correlations of CMAs.

## Proposal of the cluster-plus-glue-atom model electron counting (CPGAMEC) rule

**Figure 1:**
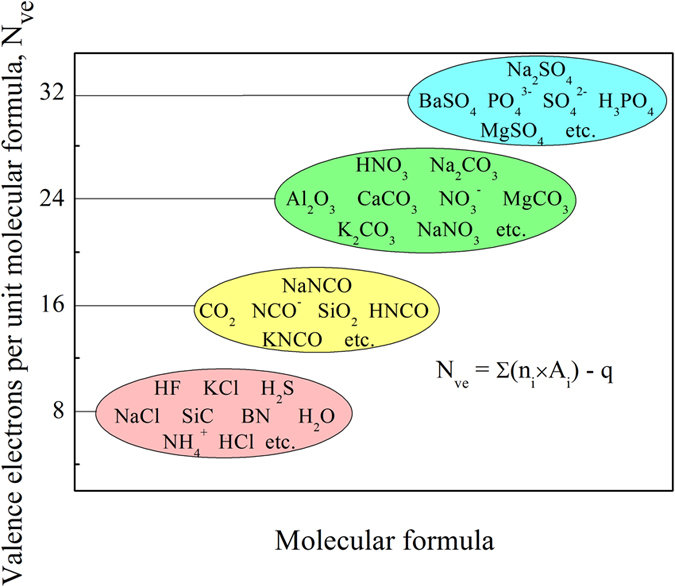
Octet rule and its extension for common covalent compounds and ionic compounds, reflected by the valence electrons’ number per unit molecule formula (*N*_*ve*_) being specific constants of eight-multiples. Chemical species related to some covalent compounds and ionic compounds are presented.

Based on the cluster-plus-glue-atom model, we set the goal of revealing the electron counting scheme for CMAs (i. e. the CPGAMEC rule), by analogy with the octet rule and its extension for common covalent compounds and ionic compounds^55^,^56^,^57^. As is known that atoms in covalent compounds and ionic compounds satisfy the octet rule by means of either sharing electrons with neighbor atoms, or transferring electrons from one atom to another^44^,^45^,^46^. As a consequence, the valence electrons’ number per unit molecular formula (*N*_*ve*_) for a majority of covalent compounds and ionic compounds is specific constants of eight-multiples^56^,^58^, like 8 for NaCl, 16 for CO_2_ and 24 for Al_2_O_3_ etc., as presented in Fig. 1 and Supplementary Table SI. Here in our work, this electron counting scheme of the *N*_*ve*_ value being constants of eight-multiples, is regarded as an extension of octet rule. Noting that it is only a sufficient condition of the octet rule, rather than its necessary condition. Accordingly, the CPGAMEC rule is proposed to reveal the electron counting scheme for CMAs, since the cluster formula of CMAs is equivalent to the molecular formula of covalent compounds and ionic compounds^15^,^29^. Analogously, the CPGAMEC rule is described by the valence electrons’ number per unit cluster formula (*N*_*e/u*_), and expressed as the following form


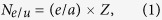


where *e/a* represents the electron concentration (i. e. the *e/a*-ratio), and *Z* represents the total number of atoms per unit cluster formula.

Given that the *e/a*-ratio has been proven to be an important concept in the theory of alloys^39^,^40^,^45^,^59^, just as the Hume-Rothery rule^47^ reflected that the structures of specific intermetallic phases (i. e. electron compounds) are stabilized by specific *e/a*-ratio (see Supplementary Figure S2). Thus, the *e/a*-ratio is used as an effective parameter to investigate the CPGAMEC rule in this work, and the methods for its calculation are presented in the following parts. Besides, the total number of atoms per unit cluster formula (*Z*) is obtained on the basis of [cluster](glue atoms)_x_. While the key point is determination of the principal cluster entering into the cluster formula: [cluster](glue atoms)_x_. To resolve this problem, an effective method of central force field model^29^,^60^ has been developed by combining interatomic force constants (*IFCs*)^61^,^62^ and atomic close packing principle^14^,^22^, while its general utility has been validated by different CMAs in numerous alloy systems^27^,^28^,^29^. For a given alloy phase, the central force field model shows that those atoms with the largest *IFCs* act as the central atom of the cluster, those atoms with the smallest *IFCs* act as the glue atoms of the model, while those atoms with the *IFCs* locating between the max. *IFCs* and min. *IFCs* act as either the shell atoms or the glue atoms^29^. Then the cutoff shell of principal cluster is determined by the atomic close-packing principle^14^,^22^, as shown in the inset map (a) of Supplementary Figure S3, the cutoff radius (*r*) of cluster shell corresponds to the maximum radial atomic density (*ρ*_*ra*_). Thereof, the principal cluster and the corresponding cluster formula can be obtained conveniently, and thus the total number of atoms per unit cluster formula (*Z*) can be achieved. Hence, the CPGAMEC rule described by the valence electrons’ number per unit cluster formula (*N*_*e/u*_), can be obtained via formula (1). In this work, the *IFCs* are computed by performing first-principles calculations within the framework of density functional perturbation theory^61^,^62^, and the computational details are provided in Supplementary Materials.

## Confirmation of the CPGAMEC rule

**Figure 2:**
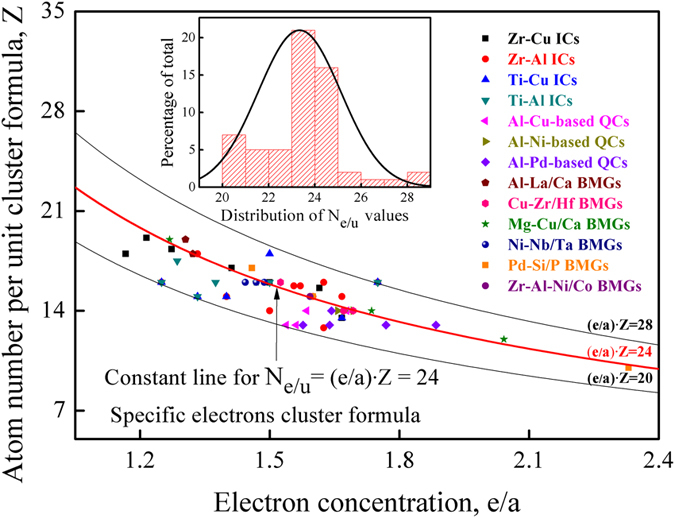
CPGAMEC rule for complex metallic alloys (including the ICs in Zr-/Ti-based systems, Al-based QCs^24^ and BMGs in several glass-forming systems^26^), reflected by the correlations between electron concentration (*e/a*) and total number of atoms per unit cluster formula (*Z*).

To confirm the existence of the CPGAMEC rule, different kinds of CMAs including Zr-/Ti-based intermetallic compounds (ICs), Al-based quasicrystals (QCs) and bulk metallic glasses (BMGs) in several glass forming systems, have been investigated by formula (1) upon analysis of their cluster structure and electron concentration information. As the results shown in Fig. 2, the *N*_*e/u*_ values of these Zr-/Ti-based ICs, Al-based QCs and BMGs, are close to specific constants of eight-multiples and twelve-multiples, verifying the existence of CPGAMEC rule for CMAs. In what follows, we will present the results and discussion about confirmation of this electron counting rule in detail.

### Confirmation of the CPGAMEC rule in ICs

**Figure 3:**
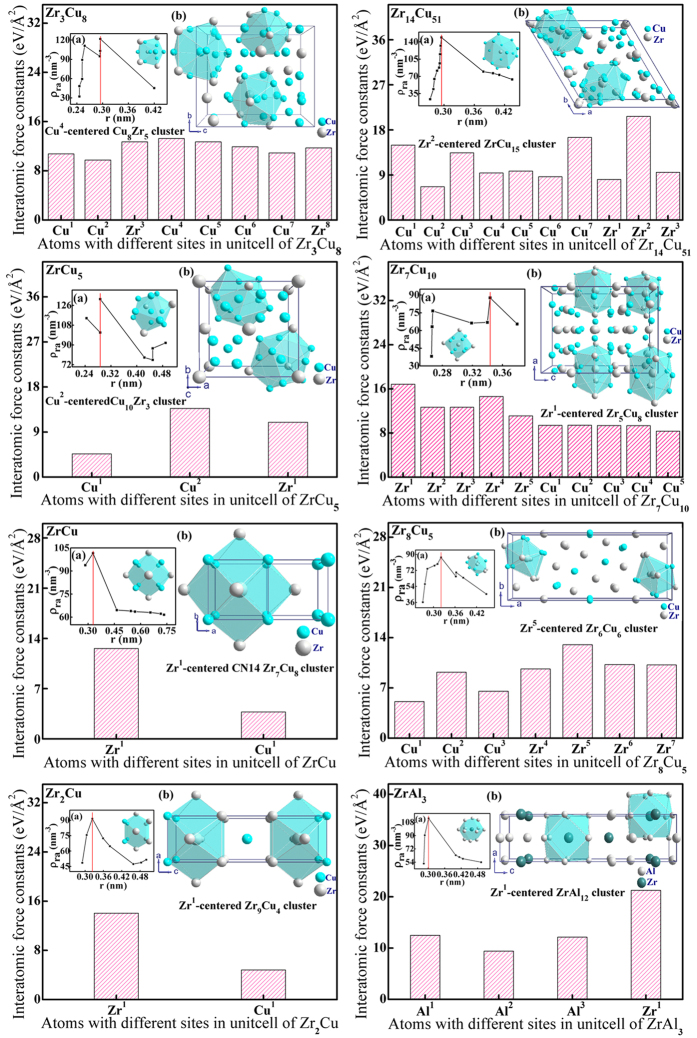
Principal clusters of the Zr-Cu/Al ICs and their interatomic force constants (*IFCs*). (**a**) Correlation between radial distances (*r*) and radial atomic density (*ρ*_*ra*_), the red vertical line depicts the cutoff radius of the principal cluster. (**b**) Atomic clusters present in the structures of Zr-Cu/Al ICs.

The Zr-Cu/Al ICs and Ti-Al/Cu ICs with apparent cluster features have been studied first^27^,^29^,^53^, and their crystallographic information are listed in Supplementary Table SII. Based on the central force field model^29^,^63^,^64^,^65^,^66^, the cluster structure information of these Zr-Cu/Al and Ti-Al/Cu ICs are obtained. The results are collected in Fig. 3, Supplementary Figure S3 and Table SIII, where the first atom represents the central atom of the principal cluster. Therewith, the total number of atoms per unit cluster formula (*Z*) for these Zr-Cu/Al ICs and Ti-Al/Cu ICs can be easily obtained. And thus the valence electrons’ number per unit cluster formula (*N*_*e/u*_) is computed via formula (1). Here the *e/a*-ratio^32^,^47^ of these Zr-Cu/Al ICs and Ti-Al/Cu ICs is calculated by weight averaging the valence electrons contribution of all constituent elements, as expressed in the following form


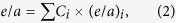


where *C*_*i*_ and (*e/a*)_*i*_ denotes the atomic fraction and the valence electrons contribution of the *i*-th element, respectively. The determination of (*e/a*)_*i*_ in those TM-containing systems, however, is complicated because of sp-d hybridization^35^,^47^,^48^,^59^, thus it is still a great challenge to completely obtain the *e/a*-ratio of these ICs. Nevertheless, the extra-nuclear electronic configuration of TMs in the periodic table is definite^67^. Besides, we notice that (*e/a*)_*i*_ assignment for the *e/a*-ratio calculation in Hume-Rothery rule is adopted as the usual valences of the constituent elements^32^,^47^,^68^. Therefore, the *e/a*-ratio of these ICs is calculated by adopting the outermost electrons and the common valences^44^ as the (*e/a*)_*i*_ assignments, respectively (see Supplementary Table SIV). The detailed discussion on the valence electrons contribution of these constituent elements is provided in Supplementary Materials.

By assigning the outermost electrons as the valence electrons contribution, the *e/a*-ratio of these ICs are calculated via formula (2). The results indicate that the *e/a*-ratio of these Zr-Cu/Al ICs and Ti-Al/Cu ICs lies within the range from 1.2 to 1.8, and it varies with the *i*-element’s content (*C*_*i*_) in a linear manner (see Supplementary Figure S4). Accordingly, the *N*_*e/u*_ values for these ICs are obtained via formula (1), and the results are presented in Table 1. It is found that in this case, the *N*_*e/u*_ value of these ICs is close to a specific constant of eight-multiples and twelve-multiples 24, as shown in Fig. 2. Meanwhile, in the case when the *e/a*-ratios of these ICs are calculated by assigning the common valences as the valence electrons contribution, the *N*_*e/u*_ value for these ICs is again found to approach a specific constant of eight-multiples and twelve-multiples 48 (see Supplementary Table SV). The results indicate that in both cases, the *N*_*e/u*_ values of these ICs are close to the specific constants of eight-multiples and twelve-multiples, as shown in Supplementary Figure S5, which confirms the existence of the CPGAMEC rule in alloy compounds, just as the extension of octet rule for covalent compounds and ionic compounds. For convenience, this electron counting scheme for CMAs is termed as the specific electrons cluster formula. For some ICs’ *N*_*e/u*_ values deviating from the specific constants of eight-multiples and twelve-multiples (see Supplementary Figure S5), it arises from the (*e/a*)_*i*_ assignment in the *e/a*-ratio calculation process.

### Confirmation of the CPGAMEC rule in QCs and BMGs

**Figure 4:**
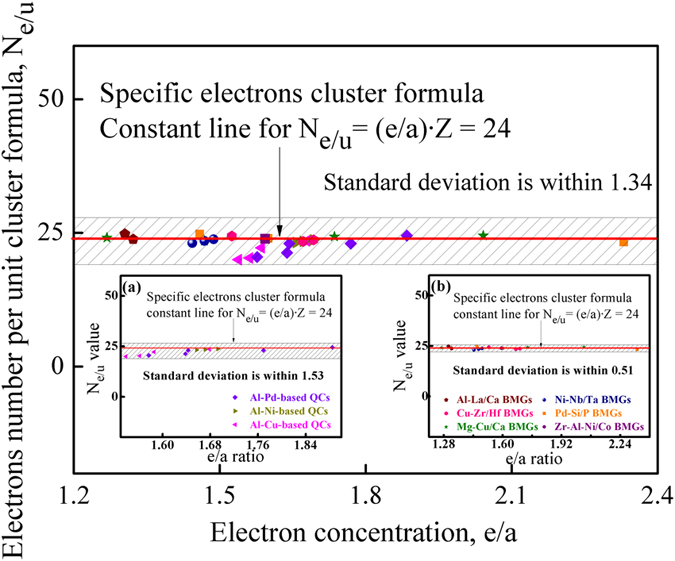
Correlation between electron concentration (*e/a*) and valence electrons’ number per unit cluster formula (*N*_*e/u*_) for typical Al-based QCs and BMGs in several glass-forming systems^28^,^29^, reflecting the CPGAMEC rule of specific electrons cluster formula for CMAs and their *N*_*e/u*_ values’ deviation from the specific constant.

Based on the cluster-resonance model^20^,^69^, it has been found that the valence electrons’ number per unit cluster formula (*N*_*e/u*_) for typical QCs and BMGs^24^,^26^, is also close to the specific constant of eight-multiples and twelve-multiples 24, as shown in Fig. 4. This coincidence implies that QCs and BMGs follow the CPGAMEC rule as well. As for those QCs and BMGs, whose structures are stabilized by the Fermi sphere-Brillouin zone interaction^70^,^71^,^72^, the cluster-resonance model provides another applicable method to calculate their *e/a*-ratio, as expressed in the following form


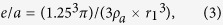


where *r*_1_ and *ρ*_*a*_ each represents the principal cluster radius and the atomic density^15^,^50^. Accordingly, the *e/a*-ratio for some Al-based QCs and typical BMGs is calculated. While the *Z* value is acquired from the cluster formula of these QCs and BMGs, which has been successfully used to explain their experimental compositions (see Supplementary Table SVI and SVII). Hence, the *N*_*e/u*_ values for these Al-based QCs and BMGs^24^,^26^ are obtained, and the results are presented in Fig. 4 and Supplementary Figure S6. As reflected that the *N*_*e/u*_ values for these QCs and BMGs are close to the specific constant of eight-multiples and twelve-multiples 24, confirming the existence of the CPGAMEC rule in QCs and BMGs. Furthermore, the *N*_*e/u*_ values’ standard deviation from constant 24 for BMGs is smaller than that for Al-based QCs (see insets in Fig. 4). This distinction is attributed to the structural differences of these CMAs, as will be discussed in the following part.

All of the results reveal the fact that the *N*_*e/u*_ values of Zr-/Ti-based ICs, Al-based QCs and typical BMGs are close to the specific constants of eight-multiples and twelve-multiples (see Fig. 2), which confirms the existence of CPGAMEC rule for CMAs. Moreover, the CPGAMEC rule signifies that CMAs’ cluster formula is superior to the customary stoichiometric formula^54^. Meanwhile, the eight-multiples’ characteristic of the *N*_*e/u*_ values for CMAs suggests that CMAs follow the extension of octet rule as well. Besides, the cluster formula of CMAs may differ from the molecular formula of covalent compounds and ionic compounds only by the linkage between the primary units^44^, which retains the basic features of interatomic interaction instead of inter-molecular forces. As for some CMAs deviating from the CPGAMEC rule, it is attributed to the *e/a*-ratios involving in the *N*_*e/u*_ calculated process, this can be readily understood since the *e/a*-ratio is only an effective one because of TMs’ hybridized effects^15^,^20^. The situation is similar to the exception of octet rule, where special terminology like hyper-/hypo-valence has been developed to describe those chemical species that do not follow the octet rule^46^,^57^. Furthermore, the CPGAMEC rule is fairly well followed by those CMAs with apparent cluster features, just as the octet rule is strictly followed by element atoms in the period two, while element atoms in other periods may obey this rule but not necessarily in all molecules^11^,^12^,^57^. Especially, the electron counting rules followed by a large number of condensed matters (including CMAs, covalent compounds and ionic compounds), imply that the valence electrons’ number per unit molecular formula is close to specific constants, which are firmly related with materials’ atomic structure. Besides, the existence of the CPGAMEC rule in ICs, QCs and BMGs further reveals the close relationship between structure and properties of these CMAs^15^,^29^,^73^,^74^.

During the past decades, some other electron counting schemes have been developed to explore the interrelationship between the structure and properties of materials^75^,^76^,^77^,^78^,^79^,^80^,^81^,^82^,^83^,^84^,^85^,^86^,^87^,^88^,^89^,^90^,^91^,^92^,^93^,^94^,^95^,^96^,^97^,^98^,^99^. For example, the skeletal electron pair (SEP) rule^76^,^77^,^78^,^79^ used to describe the cluster structural features of complex polynuclear molecules with varied skeletal atoms^85^,^86^, the topology electron counting (TEC)^80^,^82^ theory used to estimate the electron counts of polyhedral metal clusters with varying nuclearity^79^,^81^,^84^,^87^. Both SEP and TEC theories assume that each vertex atom contributes three orbitals to the cluster bonding^75^,^77^,^88^. Nevertheless, this assumption is true for the main-group elements but not necessarily true for the transition metals^87^. Besides, the hypervalent electron counting scheme and the Zintl-Klemm electron counting rule^92^,^93^, provide a route for understanding the bonding in ICs containing heavy main group elements. While the 14 electron rule^94^,^95^ indicates that the total valence electrons’ number per transition metal atom in Nowotny chimney ladder phases is 14. Compared with these electron counting schemes^75^,^76^,^77^,^78^,^79^,^80^,^81^,^82^,^83^,^84^,^85^,^86^,^87^,^88^,^89^,^90^,^91^,^92^,^93^,^94^,^95^,^96^,^97^,^98^,^99^, the CPGAMEC rule pay much attention on those CMAs with apparent cluster structural features, like some ICs, QCs and BMGs. All of these electron counting rules made significant progress in our better understanding of the close connections among the valence electrons number, the cluster stereochemistry and the atomic cluster geometries.

## Application of the CPGAMEC rule

The CPGAMEC rule can be applied to guide the composition design of CMAs with desired properties. Based on the cluster-plus-glue-atom model and the CPGAMEC rule, it is clear that this electron counting scheme endows the cluster formula of CMAs with apparent molecular features, just like the molecular formula of common covalent and ionic compounds. In this context, the cluster formula corresponding to cluster-plus-glue-atom model brings with itself the basic information on CMAs’ composition, atomic structure and electronic unit. Accordingly, the composition-structure-property correlations of CMAs can be investigated further. In the present work, we take BMGs in the ZrCu-based system as an example to explain the correlations reflected by the CPGAMEC rule, and to illustrate its practical applications in CMAs’ composition designing process.

**Figure 5:**
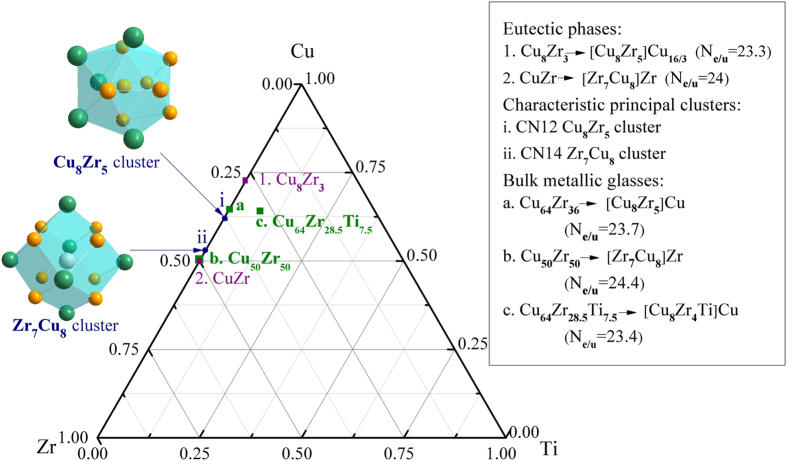
Illustration for the application of the CPGAMEC rule: Specific electrons cluster formula of CuZr-based bulk metallic glasses, and the principal clusters derived from relevant eutectic phases in Cu-Zr alloy system, reflected in ternary Cu-Zr-Ti phase diagram. (In this and subsequent figures, the orange spheres and olive green spheres denote the copper atoms and zirconium atoms, respectively).

As shown in Fig. 5, based on the Cu_8_Zr_5_ icosahedral cluster derived from Zr_3_Cu_8_ ICs^29^, the BMGs compositions can be designed via the known cluster formula of [cluster](glue atoms)_1or3_ for ideal glassy formers^15^,^19^,^21^. Then the possible cluster formulas are denoted as [Cu_8_Zr_5_]Cu, [Cu_8_Zr_5_]Zr, [Cu_8_Zr_5_]Zr_2_Cu, [Cu_8_Zr_5_]Cu_3_, [Cu_8_Zr_5_]Cu_2_Zr and [Cu_8_Zr_5_]Zr_3_. Under the theoretical guidance of CPGAMEC rule, it has been verified that among these cluster formulas, the specific electrons cluster formula [Cu_8_Zr_5_]Cu = Cu_64.3_Zr_35.7_ with its *N*_*e/u*_ = 23.7 close to the specific constant of eight-multiple and twelve multiple 24, is in good agreement with the experimentally synthesized Cu_64_Zr_36_ BMGs. Likewise, the specific electrons cluster formula [Zr_7_Cu_8_]Zr and [Ti_9_Cu_6_]Cu_3_, with *N*_*e/u*_ = 24.2 and 23.6 close to ideal value 24, can be used to explain the composition of Cu_50_Zr_50_ and Cu_50_Ti_50_ BMGs, where the Zr_7_Cu_8_ and Ti_9_Cu_6_ principal clusters are derived from ZrCu ICs and TiCu ICs, respectively^29^,^65^. By combination with the micro-alloying mechanism, multi-components BMGs’ compositions can be achieved via element substitution method^15^. For instance, when one of the shell atoms Zr in binary cluster formula [Cu_8_Zr_5_]Cu is substituted by one Ti atom with comparable size^21^, the experimental composition for ternary Cu_64_Zr_28.5_Ti_7.5_ BMGs can be designed via the specific electrons cluster formula [Cu_8_Zr_4_Ti]Cu = Cu_64.3_Zr_28.6_Ti_7.1_, with *N*_*e/u*_ = 23.4 close to the specific constant of eight-multiple and twelve-multiple 24. Likewise, the composition of quaternary Ti_40_Cu_46.95_Zr_10_Sn_3.05_ BMGs can be designed on the basis of binary specific electrons cluster formula: [Ti_9_Cu_6_]Cu_3_ via elements’ substitution method, and its resultant cluster formula is [TiCu_5.45_Sn_0.55_Ti_6.2_Zr_1.8_]Cu_3_ with *N*_*e/u*_ = 24.5 close to the specific constant of eight-multiple and twelve-multiple 24. Relevant experimental studies indicate this quaternary BMGs have good glass forming ability and high strength^65^. Therefore, the CPGAMEC rule provides an innovative theoretical guidance to direct the design of CMAs with desired properties.

## Interpretation of the CPGAMEC rule

**Figure 6:**
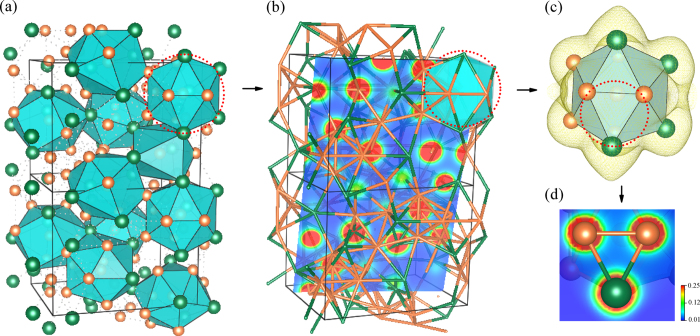
General view of the interpretation for the CPGAMEC rule, based on the charge distribution of Gauss’s law and the cluster structural features of CMAs: (**a**) Atomic clusters existed in the structure of Zr_3_Cu_8_ ICs, (**b**) Charge density distribution of Zr_3_Cu_8_ ICs, (**c**) Charge density of the *CN*12 Cu_8_Zr_5_ icosahedral cluster presented in the structure of Zr_3_Cu_8_ ICs (the isosurface is 0.02 e/Bohr^3^), (**d**) Color-contour maps on the Cu-Zr-Cu triangle plane of *CN*12 Cu_8_Zr_5_ cluster in Zr_3_Cu_8_ ICs.

To make further progress, a possible interpretation for understanding the CPGAMEC rule has been presented on the basis of CMAs’ cluster structural characteristics. As mentioned above, the *N*_*e/u*_ values for these CMAs are close to the specific constants of twelve-multiples. Meanwhile, the local atomic structures of CMAs are characterized by numerous polyhedral clusters^4^,^13^,^14^,^15^, and most of these clusters are the convex polyhedron with coordination number (*CN*) of twelve^100^,^101^,^102^,^103^. In particular, the short-range-ordering features induced by the *CN*12 icosahedral clusters in the structure of BMGs and IQCs, have been verified by many theoretical and experimental investigations^104^,^105^,^106^,^107^,^108^,^109^,^110^,^111^,^112^,^113^. On this ground, we assume the specific electrons cluster formula as an entire *CN*12 convex polyhedron, while this polyhedron contains the basic information on composition, structure and electrons of CMAs. According to the charge distribution of Gauss’s law^114^,^115^,^116^ and under the above assumption, it is readily to understand that the *N*_*e/u*_ values for these CMAs are close to the specific constants of twelve-multiples. Meanwhile, the principal cluster in the cluster formula represents CMAs’ main structural features, while the glue atoms is only a small part and can be averaged into the cluster part^15^,^20^. For instance, the *CN*12 Cu_8_Zr_5_ icosahedral cluster represents the primary structural features of Zr_3_Cu_8_ phase^15^,^29^. Figure 6 presents the atomic cluster structures and the charge density distribution of Zr_3_Cu_8_ phase, it shows that the electrons mainly distribute on the twelve vertex of Cu_8_Zr_5_ clusters, which further demonstrates the rationality of this interpretation for the CPGAMEC rule. Our understanding on the CPGAMEC rule further implies that the electron counting schemes of materials are closely related to their microscopic atomic structures.

From the viewpoint of CMAs’ atomic cluster structures, the above interpretation for the specific electrons cluster formula provides an underlying mechanism behind the CPGAMEC rule. Accordingly, the *N*_*e/u*_ values’ deviation from the specific constants of twelve-multiples for some CMAs (see the inset in Fig. 2), can be understood as distortions of the *CN*12 convex polyhedron^4^,^74^. Furthermore, the fact that the *N*_*ve*_ values for some covalent compounds and ionic compounds are specific constants of eight-multiples, is regarded as an extension of the octet rule. This can be understood as the valence electrons distribute in successive shells at the corners of a cube^38^,^57^. Similarly, the fact that the *N*_*e/u*_ values for these CMAs are specific constants of twelve-multiples, can be understood as the valence electrons distribute in successive shells at the vertexes of the *CN*12 convex polyhedron. Therefore, the CPGAMEC rule of the specific electrons cluster formula, provides an aggregate picture with intriguing electronic rule and structural features of CMAs. It is worthwhile to mention that there are other underlying mechanisms behind this CPGAMEC rule, and our studies along this direction are still underway.

In conclusion, an electron counting rule for CMAs (i. e. CPGAMEC rule) has been presented in this work, by analogy with the extension of octet rule for common covalent compounds and ionic compounds. It has been found that the valence electrons’ number per unit cluster formula (*N*_*e/u*_) for different kinds of CMAs, are close to specific constants of eight-multiples and twelve-multiples, as exemplified by Zr-/Ti-based ICs, Al-based QCs and BMGs in several glass-forming systems. Thus we termed it as CMAs’ specific electrons cluster formula. It has been demonstrated that the CPGAMEC rule is a useful guidance to direct the design of CMAs with desired properties. Meanwhile, the cluster formula can be regarded as not only CMAs’ composition unit and structural unit, but also their electronic unit and molecular formula. Furthermore, the CPGAMEC rule for CMAs imply that the electron counting schemes of materials are closely related to their atomic structure features. The present work provides an aggregate picture with intriguing electronic rule and structural features of CMAs, and hence offers a significant theoretical guidance for researchers to further investigate the composition-structure-properties correlations of CMAs.

## Additional Information

**How to cite this article**: Du, J. *et al*. Hidden electronic rule in the “cluster-plus-glue-atom” model. *Sci. Rep.* **6**, 33672; doi: 10.1038/srep33672 (2016).

## Supplementary Material

Supplementary Information

## Figures and Tables

**Table 1:** Cluster information for the Zr-Cu/Al and Ti-Cu/Al ICs, including the principal cluster with its coordination number (*CN*), cluster formula, total number of atoms per unit cluster formula (*Z*), electron concentration (*e/a*) and valence electrons’ number per unit cluster formula (*N*_*e/u*_), calculated via formula (1) with *e/a* obtained from formula (2), where the (*e/a*)_*i*_ is assigned as the outermost electrons of the i-element.

Alloy system	ICs’ composition	Principal cluster	Cluster formula	*e/a*	*Z*	*N*_*e/u*_
Zr-Cu/Al	ZrCu_5_	*CN*12 Cu_10_Zr_3_ cluster	[Cu_10_Zr_3_](Cu_5_)	1.167	18	21
	Zr_14_Cu_51_	*CN*15 ZrCu_15_ cluster	[ZrCu_15_](Zr_159/51_)	1.215	19.12	23.24
	Zr_3_Cu_8_	*CN*12 Cu_8_Zr_5_ cluster	[Cu_8_Zr_5_](Cu_16/3_)	1.273	18.33	23.33
	Zr_7_Cu_10_	*CN*12 Zr_5_Cu_8_ cluster	[Zr_5_Cu_8_](Cu_2_Zr_2_)	1.412	17	24
	ZrCu	*CN*14 Zr_7_Cu_8_ cluster	[Zr_7_Cu_8_](Zr)	1.5	16	24
	Zr_8_Cu_5_	*CN*11 Zr_6_Cu_6_ cluster	[Zr_6_Cu_6_](Zr_18/5_)	1.615	15.6	25.2
	Zr_2_Cu	*CN*12 Zr_9_Cu_4_ cluster	[Zr_9_Cu_4_](Cu_1/2_)	1.667	13.5	22.5
	ZrAl_3_	*CN*12 ZrAl_12_ cluster	[ZrAl_12_](Zr_3_)	1.25	16	20
	ZrAl_2_	*CN*16 Zr_5_Al_12_ cluster	[Zr_5_Al_12_](Zr)	1.333	18	24
	Zr_2_Al_3_	*CN*13 Zr_5_Al_9_ cluster	[Zr_5_Al_9_](Zr)	1.4	15	21
	ZrAl	*CN*13 Zr_7_Al_7_ cluster	[Zr_7_Al_7_]	1.5	14	21
	Zr_5_Al_4_	*CN*11 Zr_5_Al_7_ cluster	[Zr_5_Al_7_](Zr_15/4_)	1.556	15.75	24.51
	Zr_4_Al_3_	*CN*14 Zr_9_Al_6_ cluster	[Zr_9_Al_6_](Al_3/4_)	1.571	15.75	24.74
	Zr_3_Al_2_	*CN*14 Zr_9_Al_6_ cluster	[Zr_9_Al_6_]	1.6	15	24
	P6_3_/mmc-Zr_5_Al_3_	*CN*14 Zr_9_Al_6_ cluster	[Zr_9_Al_6_](Zr)	1.625	16	26
	I4/mcm-Zr_5_Al_3_	*CN*10 Al_3_Zr_8_ cluster	[Al_3_Zr_8_](Al_9/5_)	1.625	12.8	20.8
	Zr_2_Al	*CN*11 Zr_7_Al_5_ cluster	[Zr_7_Al_5_](Zr_3_)	1.667	15	25
	Zr_3_Al	*CN*12 Zr_9_Al_4_ cluster	[Zr_9_Al_4_](Zr_3_)	1.75	16	28
Ti-Al/Cu	TiAl_3_	*CN*12 TiAl_12_ cluster	[TiAl_12_](Ti_3_)	1.25	16	20
	Ti_2_Al_5_	*CN*12 Ti_5_Al_8_ cluster	[Ti_5_Al_8_](Al_9/2_)	1.286	17.5	22.5
	TiAl_2_	*CN*12 Ti_3_Al_10_ cluster	[Ti_3_Al_10_](Ti_2_)	1.333	15	20
	Ti_3_Al_5_	*CN*12 Ti_3_Al_10_ cluster	[Ti_3_Al_10_](Ti_3_)	1.375	16	22
	TiAl	*CN*14 Ti_7_Al_8_ cluster	[Ti_7_Al_8_](Ti)	1.5	16	24
	Ti_3_Al	*CN*12 AlTi_12_ cluster	[AlTi_12_](Al_3_)	1.75	16	28
	TiCu_3_	*CN*12 TiCu_12_ cluster	[TiCu_12_](Ti_3_)	1.25	16	20
	TiCu_2_	*CN*14 Ti_5_Cu_10_ cluster	[Ti_5_Cu_10_]	1.333	15	20
	Ti_2_Cu_3_	*CN*14 Ti_6_Cu_9_ cluster	[Ti_6_Cu_9_]	1.4	15	21
	TiCu	*CN*14 Ti_9_Cu_6_ cluster	[Ti_9_Cu_6_](Cu_3_)	1.5	18	27
	Ti_2_Cu	*CN*12 Ti_9_Cu_4_ cluster	[Ti_9_Cu_4_](Cu_1/2_)	1.667	13.5	22.5
	Ti_3_Cu	*CN*12 Ti_9_Cu_4_ cluster	[Ti_9_Cu_4_](Ti_3_)	1.75	16	28
