# Supplementary material for: Hidden electronic rule in the “cluster-plus-glue-atom” model
Source: Sci Rep. 2016 Sep 19;6:33672. doi: 10.1038/srep33672 (PMC5027558; doi:10.1038/srep33672)
Supplement: Supplementary Information [file srep33672-s1.doc]

**Supplementary Materials for**

**Hidden electronic rule in the “cluster-plus-glue-atom” model**

**Jinglian Du^a^, Chuang Dong^b^, Roderick Melnik^c, d^, Yoshiyuki** **Kawazoe^e, f^, Bin Wen^a^**

^a^ State Key Laboratory of Metastable Materials Science and Technology, Yanshan University, Qinhuangdao 066004, China

^b^Key Laboratory of Materials Modification [Dalian University Technology], Ministry of Education, Dalian 116024, China

^c^ The MS2Discovery Interdisciplinary Research Institute, Wilfrid Laurier University, 75 University Ave. West, Waterloo, Ontario, Canada N2L 3C5

^d^ BCAM-Basque Center for Applied Mathematics, E48009 Bilbao, Spain

^e^ New Industry Creation Hatchery Center, Tohoku University, 6-6-4 Aramaki-aza-Aoba, Aoba-ku, Sendai 980-8579, Japan

^f^ Institute of Thermophysics, Siberian Branch of the Russian Academy of Sciences, 1, Lavyrentyev Avenue, Novosibirsk 630090, Russia

**This file includes:**

·**Computational methods of the first-principles calculations**

·**Approaches for obtaining the *e/a*-ratio and the *N_e/u_* value of complex metallic alloys**

·**Table SI.** The designation, chemical formula, constituent element (*i*), outermost electrons’ number of i element (*n_i_*), atom numbers of i element (*A_i_*), charge numbers of radicals (*q*) and the total valence electrons’ number per unit chemical formula (*N_ve_*) for chemical species related to some covalent compounds and ionic compounds, where *N_ve_ = ∑(n_i_×A_i_) – q*.

·**Table SII.** Crystallographic information for the intermetallic compounds (ICs) in Zr-Cu/Al and Ti-Cu/Al alloy systems.

·**Table SIII.** Cluster information for the Zr-Cu/Al and Ti-Cu/Al ICs, including the non-equivalent atomic positions in the unitcell, atomic coordinates (*x, y, z*), interatomic force constants (*IFCs*), cutoff radius (*r*) of the cluster shell, primitive cluster, cluster coordination number (*CN*), principal cluster and cluster formula.

·**Table SIV.** Valence electrons contribution from constituent elements in Zr-Cu/Al and Ti-Cu/Al ICs, including the outermost electrons and the valences behaved in common oxides, chlorides and nitrides.

·**Table SV.** Zr-Cu/Al and Ti-Cu/Al ICs’ principal cluster with its coordination number (*CN*), cluster formula, total number of atoms per unit cluster formula (*Z*), electron concentration (*e/a*) and valence electrons’ number per unit cluster formula (*N_e/u_*).

·**Table SVI.** Al-based QCs’ principal cluster with its coordination number (*CN*), cluster formula, total number of atoms per unit cluster formula (*Z*), electron concentration (*e/a*) and valence electrons’ number per unit cluster formula (*N_e/u_*).

·**Table SVII.** Cluster information for typical BMGs in several glass-forming systems, including the principal cluster with its coordination number (*CN*), cluster formula, total number of atoms per unit cluster formula (*Z*), electron concentration (*e/a*) and valence electrons’ number per unit cluster formula (*N_e/u_*).

·**Figure S1.** Schematic illustration of the “cluster-plus-glue-atom” model, where the atoms are located close to the spherical-shell positions.

·**Figure S2.** Hume-Rothery alloys related electron concentration (*e/a*) and the corresponding structures.

·**Figure S3.** Principal clusters for the Zr-Al ICs, Ti-Al ICs and Ti-Cu ICs, and their interatomic force constants (*IFCs*). (a) Correlation between radial distances (*r*) and radial atomic density (*ρ_ra_*), the red solid vertical line depicts the cutoff radius of the principal cluster, (b) Atomic clusters present in the crystal structures of these Zr-Al ICs, Ti-Al ICs and Ti-Cu ICs.

·**Figure S4.** Correlation between electron concentration (*e/a*) and Zr/Ti-contents (*C_i_*) for the Zr-Cu/Al ICs and Ti-Cu/Al ICs with apparent cluster features.

·**Figure S5.** Specific electrons cluster formula for the Zr-Cu/Al and Ti-Cu/Al ICs, with the valence electrons’ number per unit cluster formula (*N_e/u_*) close to the specific constants of eight-multiples and twelve-multiples, reflected by the correlations between *N_e/u_* values and Zr/Ti-atomic percentage. It shows the *N_e/u_* values’ deviation from the specific constants of eight-multiples and twelve-multiples 24 and 48, from which the *e/a*-ratio is calculated by adopting the outermost electrons and the common valences as the valence electrons contribution (*e/a*)*_i_* from i element, respectively.

·**Figure S6.** Correlation between electron concentration (*e/a*) and the total number of atoms per unit cluster formula (*Z*) for typical Al-based QCs^28^ and BMGs in several glass-forming systems^29^, revealing the CPGAMEC rule of specific electrons cluster formula with *N_e/u_* = (*e/a*)×*Z* ≈ 24.

· **Computational methods of the first-principles calculations**

The crystallographic data of Zr-Cu/Al and Ti-Cu/Al intermetallic compounds (ICs) studied in this work are taken from Refs. 1, and the optimized lattice parameters are listed in Table SI. The first-principles calculations are performed within the framework of density functional theory (DFT), as implemented in the Vienne Ab initio Simulation Package (VASP)^2^. The generalized gradient approximation (GGA) with Perdew-Wang (PW91) parameterization are used to describe the exchange and correlation interactions^3,4^. The interactions between ions and valence electrons are modeled by the projector-augmented wave (PAW) method^5^. At the beginning of calculations, the cutoff energy testing for each alloy systems are performed to ensure the computational accuracy. The plain wave cutoff energies for total energy calculations are set as 320 eV for Zr-Cu ICs, 350 eV for Ti-Cu ICs, 300 eV for Zr-Al ICs and 350 eV for Ti-Al ICs, respectively. While those for subsequent interatomic forces and stress tensor calculations are set as 450 eV for Zr/Ti-Cu ICs and 480 eV for Zr/Ti-Al ICs, since the stress tensor and interatomic forces require a number of plane waves due to the slow convergence as a function of the plane waves number. Brillouin zone integrations are modeled by using a Monkhorst-Pack k-point mesh^6^, and the k-point mesh of each cell are sampled by 4×4×4, 8×8×2, 3×3×2, 8×8×8, 1×3×8, 3×3×4, 3×3×2, 6×6×1, 5×5×4, 3×2×4, 7×2×6, 3×3×4, 5×5×6, 3×3×4, 4×4×4, 2×2×5, 6×6×4, 6×6×6, 5×6×6, 6×3×6, 8×8×2, 8×8×4, 8×8×2, 6×6×7, 2×6×6, 6×6×3, 6×6×6, 6×6×1, 2×2×6 and 5×5×6 grids for ZrCu_5_, Zr_2_Cu, Zr_3_Cu_8_, ZrCu, Zr_8_Cu_5_, Zr_14_Cu_51_, Zr_7_Cu_10_, ZrAl_3_, ZrAl_2_, Zr_2_Al_3_, ZrAl, Zr_5_Al_4_, Zr_4_Al_3_, Zr_3_Al_2_, P6_3_/mmc-Zr_5_Al_3_, I4/mcm-Zr_5_Al_3_, Zr_2_Al, Zr_3_Al, TiCu_3_, TiCu_2_, Ti_2_Cu_3_, TiCu, Ti_2_Cu, Ti_3_Cu, TiAl_2_, TiAl_3_, TiAl, Ti_2_Al_5_, Ti_3_Al_5_ and Ti_3_Al, respectively. The total energy is converged numerically to 5×10^-7^ eV/atom with respect to electronic, ionic and unit cell degrees of freedom.

In addition, the first-principles calculations of dynamic matrix and its Fourier transform are performed by density functional perturbation theory (DFPT)^7-9^. For the DFPT calculations, the supercell of 2×2×2 is used for these ICs. The corresponding interatomic force constants (*IFCs*) are also computed within the framework of DFPT^10,11^, they are obtained via VASP and PHONOPY codes^2,12^. Furthermore, benchmark calculations are performed for the ZrCu ICs. The lattice parameters after optimization for bulk ZrCu ICs agree well with previously reported values^1^, confirming the reliability of the computational scheme used in this work.

- **Approaches for obtaining the *e/a*-ratio and the *N_e/u_* value of complex metallic alloys**

Electron concentration (*e/a*-ratio) plays an important role in exploring the structure and properties of materials^13-16^. Hume-Rothery rule indicates that there are close correlations between the *e/a*-ratio and the structure of electronic compounds^17^, i. e. specific crystal structures are stabilized by specific *e/a*-ratio (see Figure S2). When studying the conductive and thermal properties of a metal in relation to its band structure, those electrons entering the conduction band are concerned only and they are considered as the metallic valences^18^. In an alternative method, valence electrons are used to describe those electrons maintaining the metallic states^14^. However, the valence electrons for transition metals (TMs) are variable, since electrons in the highest occupied d orbital may be lost during a TM cation formation^13^. Consequently, most TMs can form more than one type of cation with variable valences, depending on how many d electrons are lost^18^.

Given that complex metallic alloys (CMAs) are usually with multi-components containing TM elements, and that TMs’ valences are far from being completely established to date^17,19^, thus it is still a great challenge to describe the valence electrons contribution (*e/a*)*_i_* from TMs and the *e/a*-ratio of these CMAs. Although it is reported that TMs’ valences change with the nature of their partners and the amount^17^, these bonding electrons are empirical in nature, and their theoretical significance is not clear so far^13^. Besides, it has been assumed that TMs’ d-shell is nearly full or half filled, and that the d-electrons make no obvious contribution to the cohesive forces in alloys^14^. This standpoint has provided qualitative explanation for many properties of TMs, such as interatomic distance, characteristic temperature and thermal expansion coefficient. Due to hybridization, the d-electrons indeed influence the band structure of metals and their alloys, and hence give variable valences to constituent elements^13,17^. As mentioned above, we can see that these treatments for the valence electrons contribution of TMs-containing alloys are not consistent. Nevertheless, the configuration of extra-nuclear electrons for each element in the periodic table is definite^20^. On the other hand, the valences of these elements usually behave as specific values^21,22^ in their common oxides, nitrides and chlorides (see Table SIV). Accordingly, in this work, we consider the calculation of the *e/a*-ratio for these ICs from two perspectives, i. e. by regarding the outermost electrons and the common valences of these metallic elements as their valence electrons contribution, respectively. Hence, the *e/a*-ratio of these ICs is computed by weight averaging the valence electrons contribution of the constituent elements^16,18^, i. e. *e/a*=*∑C_i_*×(*e/a*)*_i_*, where *C_i_* and (*e/a*)*_i_* each denotes the atomic fraction and the valence electrons concentration of the i-th element, and *∑C_i_*=100. In addition, the cluster resonance model^23-26^ provides another applicable method to calculate CMAs’ *e/a*-ratio based on the Fermi sphere-Brillouin zone interaction (FS-BZ)^13,19,27^. The formula is *e/a*=(1.25^3^*π*)/(3*ρ_a_*×*r*_1_^3^), with *r*_1_ and *ρ_a_* each representing the principal cluster radius and the atomic density, both of them can be obtained conveniently from the cluster-plus-glue-atom model. Since QCs and BMGs are the FS-BZ stabilized phases^28,29^, the *e/a*-ratio of Al-based QCs and BMGs in several glass-forming alloy systems can be calculated via this formula accordingly. Based on the principal cluster and cluster formula of these CMAs^30^, the total number of atoms per unit cluster formula (*Z*) can be easily achieved, and hence the valence electrons’ number per unit cluster formula (*N_e/u_*) of these CMAs is obtained via formula of *N_e/u_*=(*e/a*)×*Z*.

**References**

1. Villars P, Calvert LD. Pearson’s handbook of crystallographic data for intermetallic phases [M]. Materials Park (OH): ASM International, 1997.
2. Kresse G, Marsman M, Furthüller J. VASP the guide, [http://cms.mpi.Univie.ac.at/vasp/](http://cms.mpi.univie.ac.at/vasp/).
3. Perdew JP, Burke K, Ernzerhof M. Generalized Gradient Approximation Made Simple. *Physical Review Letters* **77**, 3865 (1996).
4. Payne MC, Teter MP, Allan DC, Arias TA, Joannopoulos JD. Iterative minimization techniques forab initiototal-energy calculations: molecular dynamics and conjugate gradients. *Reviews of Modern Physics* **64**, 1045-1097 (1992).
5. Blöchl PE. Projector augmented-wave method. *Physical Review B* **50**, 17953-17979 (1994).
6. Monkhorst HJ, Pack JD. Special points for Brillouin-zone integrations. *Physical Review B* **13**, 5188 (1976).
7. Baroni S, Giannozzi P, Testa A. Green’s-function approach to linear response in solids. *Physical Review Letters* **58**, 1861-1864 (1987).
8. Gonze X, Vigneron JP. Density-functional approach to nonlinear-response coefficients of solids. *Physical Review B* **39**, 13120-13128 (1989).
9. Gonze X, Allan DC, Teter MP. Dielectric tensor, effective charges, and phonons in α-quartz by variational density-functional perturbation theory. *Physical Review Letters* **68**, 3603-3606 (1992).
10. Gonze X, Lee C. Dynamical matrices, Born effective charges, dielectric permittivity tensors, and interatomic force constants from density-functional perturbation theory. *Physical Review B* **55**, 10355 (1997).
11. Gonze X. First-principles responses of solids to atomic displacements and homogeneous electric fields: Implementation of a conjugate-gradient algorithm. *Physical Review B* **55**, 10337 (1997).
12. Togo A, Oba F, Tanaka I. First-principles calculations of the ferroelastic transition between rutile-type and CaCl_2_-type SiO_2_ at high pressures. *Physical Review B* **78**, 134106 (2008).
13. Massalski TB, King HW. Alloy Phases of the Noble Metals. *Progress in Materials Science* **10**, 3-78 (1963).
14. Pauling L. The Nature of the Interatomic Forces in Metals. *Physical Review* **54**, 899-904 (1938).
15. Condon EU, Mack JE. An Interpretation of Pauli's Exclusion Principle. *Physical Review* **35**, 579-582 (1930).
16. Sato H, Inukai M, Zijlstra ES, Mizutani U. NFEapproximation for the e/a determination for 3d-transition metal elements and their intermetallic compounds with Al and Zn. *Philosophical Magazine* **93**, 3029-3061 (2013).
17. Raynor GV. Progress in the Theory of Alloys. *Progress in Metal Physics* **1**, 1-76 (1949).
18. Pauling L, Ewing FJ. The Ratio of Valence Electrons to Atoms in Metals and Intermetallic Compounds. *Reviews of Modern Physics* **20**, 112-122 (1948).
19. Trambly de Laissardière G, Manh DN, Magaud L, Julien JP, Cyrot-Lackmann F, Mayou D. Electronic structure and hybridization effects in Hume-Rothery alloys containing transition elements. *Physical Review B* **52**, 7920-7933 (1995).
20. Schaeffer B. IUPAC Periodic Table Quantum Mechanics Consistent. *Journal of Modern Physics* **05**, 117-122 (2014).
21. Pauling L. Influence of valence, electronegativity, atomic radii, and crest-trough interaction with phonons on the high-temperature copper oxide superconductors. *Physical Review Letters* **59**, 225-227 (1987).
22. Pauling L. On the nature of the bonding in Cu2—a Comment. *The Journal of Chemical Physics* **78**, 3346-3346 (1983).
23. Han G*, et al.* The e/a values of ideal metallic glasses in relation to cluster formulae. *Acta Materialia* **59**, 5917-5923 (2011).
24. Han G*, et al.* Composition formulae of ideal metallic glasses and their relevant eutectics established by a cluster-resonance model. *Philosophical Magazine* **91**, 2404-2418 (2011).
25. Dong C*, et al.* From clusters to phase diagrams: composition rules of quasicrystals and bulk metallic glasses. *Journal of Physics D: Applied Physics* **40**, R273-R291 (2007).
26. Häussler P*, et al.* On the formation of structure and electronic transport. *Journal of Physics and Chemistry of Solids* **68**, 753-757 (2007).
27. Stiehler M, Rauchhaupt J, Giegengack U, Häussler P. On modifications of the well-known Hume-Rothery rules: Amorphous alloys as model systems. *Journal of Non-Crystalline Solids* **353**, 1886-1891 (2007).
28. Chen H, Qiang JB, Wang YM, Dong C. Compositions of Al-Based Quasicrystals Interpreted by Cluster Formula. *Acta Physica Polonic A* **126**, 446 (2014).
29. Luo LJ*, et al.* 24 electron cluster formulas as the ‘molecular’ units of ideal metallic glasses. *Philosophical Magazine* **94**, 2520-2540 (2014).
30. Du JL, Wen B, Melnik R, Kawazoe Y. Determining characteristic principal clusters in the “cluster-plus-glue-atom” model. *Acta Materialia* **75**, 113-121 (2014).
31. Balanetskyy S, Grushko B. A study of the high-Al part of the Al-Pd-Re phase diagram. *Journal of Alloys and Compounds* **456**, 105-112 (2008).
32. Tsai A, Inoue A, Yokoyama Y, Masumoto T. Stable Icosahedral Al-Pd-Mn and Al-Pd-Re Alloys. *Materials Transactions JIM* **31**, 98-103 (1990).
33. Pavlyuchkov D, Grushko B, Velikanova TY, Al-rich region of Al-Pd-Ru at 1000 to 1100^◦^C. *Journal of Alloys and Compounds* **464**, 101-106 (2008).
34. Tsai AP, Inoues A, Yokoyam Y, Masumoto T. New icosahedral alloys with superlattice order in the Al-Pd-Mn system prepared by rapid solidification. *Philosophical Magazine Letters* **61**, 9-14 (1990).
35. Boudard M, Bourgeat-Lami E, Boissieu M., et al. Production and characterization of single quasicrystals of the Al-Pd-Mn icosahedral phase. *Philosophical Magazine Letters* **71**, 11-19 (1995).
36. Grushko B, Velikanova T. Formation of quasiperiodic and related periodic intermetallics in alloy systems of aluminum with transition metals. *Calphad* **31**, 217-232 (2007).
37. Grushko B, Lemmerz U, Fischer K, et al. The Low Temperature Instability of the Decagonal Phase in Al-Ni-Fe. *Physica Status Solidi (a)* **155**, 17-30 (1996).
38. Grushko B, Kowalski W, Przepiórzyński B, Pavlyuchkov D. Constitution of the high-Al region of Al-Cu-Rh. *Journal of Alloys and Compounds* **464**, 227-233 (2008).
39. Taniguchi S, Abe E. Highly-perfect decagonal quasicrystalline Al_64_Cu_22_Co_14_ with non-centrosymmetry. *Philosophical Magazine* **88**, 1949-1958 (2008).
40. Kamo T, Watanabe Y, Nanao S. Formation of millimeter-sized Al-Cu-Ru single quasicrystals. *Materials Transactions-JIM* **38**, 1116-1118 (1997).
41. Gratias D, Calvayrac Y, Devaud-Rzepski J, Faudot F, Harmelin M, Quivy A, Bancel PA. The phase diagram and structures of the ternary AlCuFe system in the vicinity of the icosahedral region. *Journal of Non-Crystalline Solids* **153**, 482-488 (1993).
42. Egami T, Universal criterion for metallic glass formation. *Materials Science and Engineering A* **226-228**, 261-267 (1997).
43. Wang Q, Qiang J, Wang Y, Xia J, Dong C. Bulk metallic glass formation in Cu-Zr-Ti ternary system. *Journal of Non-Crystalline Solids* **353**, 3425-3428 (2007).
44. Xia L, Ding D, Shan S, Dong Y. The glass forming ability of Cu-rich Cu-Hf binary alloys. *Journal of Physics: Condensed Matter* **18**, 3543-3548 (2006).
45. Xia L, Li W, Fang S, Wei B, Dong Y. Binary Ni-Nb bulk metallic glasses. *Journal of Applied Physics* **99**, 026103 (2006).
46. Zhu Z, Zhang H, Ding B, Hu Z. Synthesis and properties of bulk metallic glasses in the ternary Ni-Nb-Zr alloy system. *Materials Science and Engineering A* **492**, 221-229 (2008).
47. Yuan L, Pang C, Wang Y, Wang Q, Qiang J, Dong C. Understanding the Ni-Nb-Zr BMG composition from a binary eutectic Ni-Nb icosahedral cluster. *Intermetallics* **18**, 1800-1802 (2010).
48. Wang Y, Wang Q, Zhao J, Dong C. Ni-Ta binary bulk metallic glasses. *Scripta Materialia* **63**, 178-180 (2010).
49. Guo F, Poon S, Shiflet G. CaAl-based bulk metallic glasses with high thermal stability. *Applied Physics Letters* **84**, 37 (2004).
50. Chason E, Greer A, Kelton K, Pershan P, Sorensen L, Spaepen F, Weiss A. Structural relaxation of amorphous Pd_82_Si_18_: X-raymeasurements, electrical-resistivity measurements, and a comparison using the Ziman theory. *Physical Review B* **32**, 3399 (1985).
51. Plummer J, Figueroa I, Hand R, Davies H, Todd I. Elastic properties of some bulk metallic glasses. *Journal of Non-Crystalline Solids* **355**, 335-339 (2009).
52. Ma H, Shi L, Xu J, Li Y, Ma E. Discovering inch-diameter metallic glasses in three-dimensional composition space. *Applied Physics Letters* **87**, 181915 (2005).
53. Tan H, Zhang Y, Ma D, Feng Y, Li Y. Optimum glass formation at off-eutectic composition and its relation to skewed eutectic coupled zone in the La based La-Al-(Cu,Ni) pseudo ternary system. *Acta Materialia* **51**, 4551-4561 (2003).
54. Senkov O, Miracle D, Keppens V, Liaw P. Development and Characterization of Low-Density Ca-Based Bulk Metallic Glasses: An Overview. *Metallurgical and Materials Transactions A* **39,** 1888 (2008).
55. Drehman AJ, Greer AL, Turnbull D. Bulk formation of a metallic glass: Pd_40_Ni_40_P_20_. *Applied Physics Letters* **41**, 716 (1982).
56. Li FW, Qiang JB, Wang YM, Wang Q, Dong XL, Dong C. Revisiting Al-Ni-Zr bulk metallic glasses using the ‘cluster-resonance’ model. *Chinese Science Bulletin* **56**, 3902-3907 (2011).
57. Wada T, Qin F, Wang X, Yoshimura M, Inoue A, Sugiyama N, Ito R, Matsushita N. Formation and bioactivation of Zr-Al-Co bulk metallic glasses. *Journal of Materials Research* **24**, 2941 (2009).

**Table SI.** The designation, chemical formula, constituent element (*i*), outermost electrons’ number of i element (*n_i_*), atom numbers of i element (*A_i_*), charge numbers of radicals (*q*) and the total valence electrons’ number per unit chemical formula (*N_ve_*) for chemical species related to some covalent compounds and ionic compounds, where *N_ve_ = ∑(n_i_×A_i_) – q*.

| Designation | Chemical formula | i | n_i_ | A_i_ | q | N_ve_ |
| --- | --- | --- | --- | --- | --- | --- |
| Water molecule | H_2_O | H | 1 | 2 | 0 | 8 |
|  |  | O | 6 | 1 |  |  |
| Sodium chloride molecule | NaCl | Na | 1 | 1 | 0 | 8 |
|  |  | Cl | 7 | 1 |  |  |
| Ammonia ion | NH_4_^+^ | N | 5 | 1 | +1 | 8 |
|  |  | H | 1 | 4 |  |  |
| Boron nitride molecule | BN | B | 3 | 1 | 0 | 8 |
|  |  | N | 5 | 1 |  |  |
| Carborundum molecule | SiC | Si | 4 | 1 | 0 | 8 |
|  |  | C | 4 | 1 |  |  |
| Silica molecule | SiO_2_ | Si | 4 | 1 | 0 | 16 |
|  |  | O | 6 | 2 |  |  |
| Cyanate ion | NCO^-^ | N | 5 | 1 | -1 | 16 |
|  |  | C | 4 | 1 |  |  |
|  |  | O | 6 | 1 |  |  |
| Carbon dioxide molecule | CO_2_ | C | 4 | 1 | 0 | 16 |
|  |  | O | 6 | 2 |  |  |
| Alumina molecule | Al_2_O_3_ | Al | 3 | 2 | 0 | 24 |
|  |  | O | 6 | 3 |  |  |
| Carbonate ion | CO_3_^2-^ | C | 4 | 1 | -2 | 24 |
|  |  | O | 6 | 3 |  |  |
| Calcium carbonate molecule | CaCO_3_ | Ca | 2 | 1 | 0 | 24 |
|  |  | C | 4 | 1 |  |  |
|  |  | O | 6 | 3 |  |  |
| Nitrate ion | NO_3_^-^ | N | 5 | 1 | -1 | 24 |
|  |  | O | 6 | 3 |  |  |
| Vitriol molecule | H_2_SO_4_ | H | 1 | 2 | 0 | 32 |
|  |  | S | 6 | 1 |  |  |
|  |  | O | 6 | 4 |  |  |
| Sulfate ion | SO_4_^2-^ | S | 6 | 1 | -2 | 32 |
|  |  | O | 6 | 4 |  |  |
| Barium sulfate molecule | BaSO_4_ | Ba | 2 | 1 | 0 | 32 |
|  |  | S | 6 | 1 |  |  |
|  |  | O | 6 | 4 |  |  |
| Phosphate ion | PO_4_^3-^ | P | 5 | 1 | -3 | 32 |
|  |  | O | 6 | 4 |  |  |
| Phosphorus pentoxide | P_2_O_5_ | P | 5 | 2 | 0 | 40 |

**Table SII.** Crystallographic information for the intermetallic compounds (ICs) in Zr-Cu/Al and Ti-Cu/Al alloy systems.

| Phase | Space group | Pearson Symbol | Prototype | Unit cell lattice parameters (nm) | | |
| --- | --- | --- | --- | --- | --- | --- |
| ZrCu_5_ | $\text{F}\bar{4}3m$ | cF24 | AuBe_5_ | a=0.6916 |  |  |
| Zr_3_Cu_8_ | Pnma | oP44 | Hf_3_Cu_8_ | a=0.7910 | b=0.8216 | c=1.0032 |
| Zr_7_Cu_10_ | Aba2 | oC68 | Zr_7_Ni_10_ | a=0.9404 | b=0.9364 | c=1.2756 |
| ZrCu | $\text{P}m\bar{3}m$ | cP2 | CsCl | a=0.3280 |  |  |
| Zr_8_Cu_5_ | Pbam | o*26 | Al_2_Bi_6_Ca_5_ | a=1.9865 | b=0.7702 | c=0.3209 |
| Zr_14_Cu_51_ | P6/m | - | Gd_14_Ag_51_ | a=1.1454 |  | c=0.8260 |
| Zr_2_Cu | I4/mmm | tI6 | Si_2_Mo | a=0.3236 | b=1.1204 |  |
| ZrAl_3_ | I4/mmm | tI16 | ZrAl_3_ | a=0.4015 |  | c=1.7369 |
| ZrAl_2_ | P6_3_/mmc | hP12 | MgZn_2_ | a=0.5301 |  | c=0.8755 |
| Zr_2_Al_3_ | Fdd2 | oF40 | Zr_2_Al_3_ | a=0.9637 | b=1.3975 | c=0.5576 |
| ZrAl | Cmcm | oC8 | BCr | a=0.3328 | b=1.0973 | c=0.4316 |
| Zr_5_Al_4_ | P6_3_/mmc | hP18 | Ti_5_Ga_4_ | a=0.8455 |  | c=0.5803 |
| Zr_4_Al_3_ | $P\bar{6}$ | hP7 | Zr_4_Al_3_ | a=0.5441 |  | c=0.5411 |
| Zr_3_Al_2_ | P4_2_/mnm | tP20 | Zr_3_Al_2_ | a=0.7671 |  | c=0.6970 |
| Zr_5_Al_3_ | P6_3_/mmc | hP16 | Mn_5_Si_3_ | a=0.8326 |  | c=0.5548 |
| Zr_5_Al_3_ | I4/mcm | tI32 | W_5_Si_3_ | a=1.1067 |  | c=0.5395 |
| Zr_2_Al | P6_3_/mmc | hP6 | Ni_2_In | a=0.4914 |  | c=0.5915 |
| Zr_3_Al | $Pm\bar{3}m$ | cP4 | Cu_3_Au | a=0.4384 |  |  |
| TiCu_3_ | Pmmn | oP8 | TiCu_3_ | a=0.5443 | b=0.4322 | c=0.4434 |
| TiCu_2_ | Amm2 | oC12 | VAu_2_ | a=0.4416 | b=0.7970 | c=0.4576 |
| Ti_2_Cu_3_ | I4/mmm | tI10 | Os_2_Al_3_ | a=0.3135 |  | c=1.4164 |
| TiCu | P4/nmm | tP4 | TiCu | a=0.3114 |  | c=0.5914 |
| Ti_2_Cu | I4/mmm | tI6 | Si_2_Mo | a=0.2904 |  | c=1.0890 |
| Ti_3_Cu | P4/mmm | tP4 | Ti_3_Cu | a=0.4422 |  | c=0.3177 |
| TiAl_2_ | Cmmm | oC12 | ZrGa_2_ | a= 12.1622 | b= 3.9333 | c= 3.9948 |
| TiAl_3_ | I4/mmm | tI8 | TiAl_3_ | a= 3.8461 |  | c= 8.6086 |
| TiAl | P4/mmm | tP4 | AuCu | a= 3.3968 |  | c= 2.8204 |
| Ti_2_Al_5_ | P4/mmm | tP28 | Ti_2_Al_5_ | a= 3.9136 |  | c= 28.9792 |
| Ti_3_Al_5_ | Pmmm | oP4 | Ti_3_Al_5_ | a= 11.2621 |  | c= 4.0369 |
| Ti_3_Al | P6_3_/mmc | hP8 | SnNi_3_ | a= 5.7327 |  | c= 4.6351 |

**Table SIII.** Cluster information for the Zr-Cu/Al and Ti-Cu/Al ICs, including the non-equivalent atomic positions in the unitcell, atomic coordinates (*x, y, z*), interatomic force constants (*IFCs*), cutoff radius (*r*) of the cluster shell, primitive cluster, cluster coordination number (*CN*), principal cluster and cluster formula. (The superscript in primitive cluster denotes the atomic position, while the subscript denotes the number of this kind of atoms.)

| ICs | Sites | Wyck. | (*x, y, z*) | *IFCs* (eV/Å^2^) | Primitive cluster (R_0_-R_1_-R_2_-R_3_) | *r* (nm) | *CN* | Principal cluster | Cluster formula |
| --- | --- | --- | --- | --- | --- | --- | --- | --- | --- |
| ZrCu_5_ | Cu^1^ | 4c | (0.25, 0.25, 0.25) | 4.64 | Cu^1^Cu^2^_12_Zr^1^_4_ | 0.2995 | 16 |  |  |
|  | Cu^2^ | 16e | (0.625, 0.625, 0.625) | 13.72 | Cu^2^Cu^2^_6_Cu^1^_3_Zr^1^_3_ | 0.2867 | 12 | Cu^2^-centered Cu_10_Zr_3_ cluster | [Cu_10_Zr_3_](Cu_5_) |
|  | Zr^1^ | 4a | (0, 0, 0) | 10.95 | Zr^1^Cu^2^_12_Cu^1^_4_ | 0.2995 | 16 |  |  |
| Zr_3_Cu_8_ | Cu^1^ | 8d | (0.198, 0.498, 0.406) | 10.75 | Cu^1^Cu^4^_1_Cu^6^_1_Cu^7^_1_Cu^5^_1_Cu^2^_3_Zr^8^_2_Zr^3^_3_ | 0.3044 | 12 |  |  |
|  | Cu^2^ | 8d | (0.379, 0.576, 0.160) | 9.76 | Cu^2^Cu^5^_2_Cu^4^_1_Cu^1^_3_Zr^3^_2_Zr^8^_2_Cu^6^_1_Cu^2^_1_Cu^7^_1_ | 0.3003 | 13 |  |  |
|  | Zr^3^ | 8d | (0.062, 0.446, 0.140) | 12.72 | Zr^3^Cu^2^_2_Cu^7^_2_Cu^5^_1_Cu^6^_2_Cu^1^_2_Cu^4^_2_ | 0.2946 | 11 |  |  |
|  | Cu^4^ | 4c | (0.323, 0.25, 0.516) | 13.26 | Cu^4^Cu^7^_1_Cu^6^_1_Cu^5^_1_Cu^1^_2_Cu^2^_2_Zr^3^_4_Zr^8^_1_ | 0.2944 | 12 | Cu^4^-centered Cu_8_Zr_5_ cluster | [Cu_8_Zr_5_](Cu_16/3_) |
|  | Cu^5^ | 4c | (0.367, 0.25, 0.765) | 12.70 | Cu^5^Cu^6^_2_Cu^4^_1_Cu^1^_2_Cu^2^_4_Zr^3^_2_Zr^8^_1_ | 0.2910 | 12 |  |  |
|  | Cu^6^ | 4c | (0.116, 0.25, 0.903) | 11.92 | Cu^6^Cu^5^_2_Cu^4^_1_Cu^1^_2_Cu^2^_2_Zr^3^_4_Zr^8^_1_ | 0.3072 | 12 |  |  |
|  | Cu^7^ | 4c | (0.285, 0.25, 0.273) | 10.91 | Cu^7^Cu^4^_1_Cu^1^_2_Zr^8^_2_Zr^3^_4_Cu^2^_2_ | 0.3146 | 11 |  |  |
|  | Zr^8^ | 4c | (0.459, 0.25, 0.047) | 11.73 | Zr^8^Cu^7^_2_Cu^1^_4_Cu^2^_4_Cu^5^_1_Cu^4^_1_Cu^6^_1_ | 0.3146 | 13 |  |  |
| Zr_8_Cu_5_ | Cu^1^ | 2c | (0, 0.5, 0) | 5.11 | Cu^1^Zr^5^_4_Zr^6^_4_Cu^1^_2_Zr^4^_2_ | 0.3374 | 12 |  |  |
|  | Cu^2^ | 4g | (0.706, 0.789, 0.) | 9.18 | Cu^2^Zr^5^_2_Zr^7^_4_Cu^3^_1_Zr^4^_1_ | 0.2879 | 8 |  |  |
|  | Cu^3^ | 4g | (0.095, 0.928, 0) | 6.55 | Cu^3^Cu^2^_1_Zr^6^_4_Zr^5^_2_Zr^7^_2_Cu^3^_2_Zr^4^_1_ | 0.3267 | 12 |  |  |
|  | Zr^4^ | 4g | (0.844, 0.676, 0) | 9.65 | Zr^4^Cu^2^_2_Zr^5^_2_Zr^6^_2_Zr^7^_4_Zr^4^_2_Cu^3^_1_Cu^1^_1_ | 0.3384 | 14 |  |  |
|  | Zr^5^ | 4h | (0.898, 0.373, 0.5) | 13.02 | Zr^5^Cu^2^_2_Cu^1^_2_Cu^3^_2_Zr^4^_2_Zr^6^_1_Zr^5^_2_ | 0.3210 | 11 | Zr^5^-centered Zr_6_Cu_6_ cluster | [Zr_6_Cu_6_](Zr_18/5_) |
|  | Zr^6^ | 4h | (0.537, 0.314, 0.5) | 10.25 | Zr^6^Cu^3^_4_Cu^1^_2_Zr^4^_2_Zr^5^_1_Zr^6^_3_ | 0.3217 | 12 |  |  |
|  | Zr^7^ | 4h | (0.288, 0.502, 0.5) | 10.20 | Zr^7^Cu^2^_4_Cu^3^_2_Zr^4^_4_Zr^7^_2_ | 0.3384 | 12 |  |  |
| Zr_7_Cu_10_ | Cu^1^ | 8b | (0.210, 0.001, 0.758) | 9.40 | Cu^1^Cu^2^_1_Zr^4^_2_Zr^2^_1_Zr^3^_1_Cu^4^_2_Cu^5^_1_Zr^1^_1_Zr^5^_1_ | 0.3206 | 10 |  |  |
|  | Cu^2^ | 8b | (0.292, 0.008, 0.596) | 9.42 | Cu^2^Cu^1^_1_Zr^1^_1_Cu^5^_1_Zr^4^_2_Cu^3^_2_Zr^5^_1_Zr^2^_1_Zr^3^_1_ | 0.2846 | 10 |  |  |
|  | Cu^3^ | 8b | (0.003, 0.292, 0.598) | 9.37 | Cu^3^Zr^4^_2_Zr^1^_1_Cu^5^_1_Cu^2^_2_Zr^5^_1_Zr^2^_1_Zr^3^_1_Cu^4^_1_ | 0.2818 | 10 |  |  |
|  | Cu^4^ | 8b | (0.012, 0.298, 0.308) | 9.34 | Cu^4^Zr^1^_1_Zr^4^_2_Cu^5^_1_Cu^1^_2_Cu^3^_1_Zr^5^_1_Zr^2^_1_Zr^3^_1_ | 0.2881 | 10 |  |  |
|  | Cu^5^ | 8b | (0.106, 0.109, 0.450) | 8.33 | Cu^5^Cu^4^_1_Zr^5^_3_Cu^2^_1_Cu^3^_1_Zr^3^_1_Zr^2^_1_Cu^5^_1_ | 0.2896 | 9 |  |  |
|  | Zr^1^ | 4a | (0, 0, 0.954) | 16.84 | Zr^1^Cu^4^_2_Cu^2^_2_Cu^3^_2_Cu^1^_2_Zr^5^_4_ | 0.3436 | 12 | Zr^1^-centered Zr_5_Cu_8_ cluster | [Zr_5_Cu_8_](Cu_2_Zr_2_) |
|  | Zr^2^ | 4a | (0, 0, 0.643) | 12.68 | Zr^2^Cu^1^_2_Cu^3^_2_Cu^2^_2_Cu^4^_2_Cu^5^_2_ | 0.2849 | 10 |  |  |
|  | Zr^3^ | 4a | (0, 0, 0.257) | 12.68 | Zr^3^Cu^1^_2_Cu^3^_2_Cu^5^_2_Cu^2^_2_Cu^4^_2_ | 0.2873 | 10 |  |  |
|  | Zr^4^ | 8b | (0.246, 0.256, 0.702) | 14.63 | Zr^4^Cu^1^_2_Cu^3^_2_Cu^4^_2_Cu^2^_2_ | 0.2827 | 8 |  |  |
|  | Zr^5^ | 8b | (0.313, 0.311, 0.453) | 11.11 | Zr^5^Cu^5^_3_Cu^3^_1_Cu^2^_1_Cu^4^_1_ | 0.2896 | 6 |  |  |

Table SIII. continued

| ICs | Sites | Wyck. | (*x, y, z*) | *IFCs* (eV/Å^2^) | Primitive cluster (R_0_-R_1_-R_2_-R_3_) | *r* (nm) | *CN* | Principal cluster | Cluster formula |
| --- | --- | --- | --- | --- | --- | --- | --- | --- | --- |
| Zr_2_Cu | Cu^1^ | 2a | (0, 0, 0) | 4.82 | Cu^1^Zr^1^_8_Cu^1^_4_ | 0.3236 | 12 |  |  |
|  | Zr^1^ | 4e | (0, 0, 0.34) | 14.04 | Zr^1^Cu^1^_4_Zr^1^_8_ | 0.3236 | 12 | Zr^1^-cnetered Zr_9_Cu_4_ cluster | [Zr_9_Cu_4_](Cu_1/2_) |
| ZrCu | Cu^1^ | 1a | (0, 0, 0) | 3.76 | Cu^1^Zr^1^_8_Cu^1^_6_ | 0.3280 | 14 |  |  |
|  | Zr^1^ | 1b | (0.5, 0.5, 0.5) | 12.59 | Zr^1^Cu^1^_8_Zr^1^_6_ | 0.3280 | 14 | Zr^1^-centered Zr_7_Cu_8_ cluster | [Zr_7_Cu_8_](Zr) |
| Zr_14_Cu_51_ | Cu^1^ | 2c | (0.333, 0.667, 0) | 14.96 | Cu^2^Cu^1^_1_Cu^5^_3_Cu^6^_3_Zr^3^_3_ | 0.2903 | 11 |  |  |
|  | Cu^2^ | 4h | (0.333, 0.667, 0.293) | 6.66 | Cu^2^Cu^1^_1_Cu^5^_3_Cu^6^_3_Zr^3^_3_ | 0.2799 | 10 |  |  |
|  | Cu^3^ | 6k | (0.061, 0.240, 0.5) | 13.42 | Cu^3^Cu^3^_2_Cu^6^_2_Cu^4^_4_Zr^3^_2_Zr^1^_2_ | 0.2969 | 12 |  |  |
|  | Cu^4^ | 12l | (0.191, 0.265, 0.236) | 9.41 | Cu^4^Cu^7^_2_Cu^5^_1_Cu^3^_2_Cu^6^_2_Cu^4^_2_Zr^1^_1_Zr^2^_2_Zr^3^_1_ | 0.3013 | 13 |  |  |
|  | Cu^5^ | 12l | (0.494, 0.116, 0.152) | 9.78 | Cu^5^Cu^5^_2_Cu^4^_1_Cu^6^_2_Cu^2^_1_Cu^1^_1_Zr^2^_3_Zr^3^_1_ | 0.2934 | 11 |  |  |
|  | Cu^6^ | 12l | (0.104, 0.437, 0.329) | 8.64 | Cu^6^Cu^3^_1_Cu^5^_2_Cu^4^_2_Cu^2^_1_Zr^3^_3_Zr^2^_1_Cu^6^_1_ | 0.2856 | 11 |  |  |
|  | Cu^7^ | 6j | (0.114, 0.135, 0) | 16.49 | Cu^7^Cu^7^_5_Cu^4^_4_Zr^2^_2_Zr^1^_2_ | 0.2950 | 13 |  |  |
|  | Zr^1^ | 2e | (0, 0, 0.311) | 8.08 | Zr^1^Cu^4^_6_Cu^3^_6_Cu^7^_6_ | 0.2950 | 18 |  |  |
|  | Zr^2^ | 6j | (0.114, 0.389, 0) | 20.71 | Zr^2^Cu^7^_2_Cu^6^_2_Cu^4^_4_Cu^5^_6_Cu^1^_1_ | 0.2949 | 15 | Zr^2^-centered ZrCu_15_ cluster | [ZrCu_15_](Zr_159/15_) |
|  | Zr^3^ | 6k | (0.471, 0.142, 0.5) | 9.53 | Zr^3^Cu^6^_6_Cu^2^_2_Cu^3^_2_Cu^5^_2_Cu^4^_2_ | 0.3013 | 14 |  |  |
| ZrAl_3_ | Al^1^ | 4c | (0, 0.5, 0) | 12.49 | Al^1^Al^1^_4_Zr^1^_4_Al^3^_4_ | 0.3059 | 12 |  |  |
|  | Al^2^ | 4d | (0, 0.5, 0.25) | 9.41 | Al^2^Al^2^_4_Al^3^_4_Zr^1^_4_ | 0.30135 | 12 |  |  |
|  | Al^3^ | 4e | (0, 0, 0.366) | 12.15 | Al^3^Al^2^_4_Zr^1^_4_Al^1^_4_ | 0.3059 | 12 |  |  |
|  | Zr^1^ | 4e | (0, 0, 0.119) | 21.25 | Zr^1^Al^3^_4_Al^1^_4_Al^2^_4_ | 0.3014 | 12 | Zr^1^-centered ZrAl_12_ cluster | [ZrAl_12_](Zr_3_) |
| ZrAl_2_ | Al^1^ | 2a | (0, 0, 0) | 13.03 | Al^1^Al^2^_6_Zr^1^_2_Zr^1^_4_ | 0.3102 | 12 |  |  |
|  | Al^2^ | 6h | (0.828, 0.656, 0.25) | 14.23 | Al^2^Al^2^_2_Al^1^_2_Al^2^_2_Zr^1^_6_ | 0.3127 | 12 |  |  |
|  | Zr^1^ | 4f | (0.333, 0.667, 0.065) | 15.08 | Zr^1^Al^2^_6_Al^1^_3_Al^2^_3_Zr^1^_4_ | 0.3256 | 16 | Zr^1^-centered Zr_5_Al_12_ cluster | [Zr_5_Al_12_](Zr) |
| Zr_2_Al_3_ | Al^1^ | 8a | (0, 0, 0.62) | 12.42 | Al^1^Al^2^_2_Zr^1^_4_Al^2^_2_Zr^1^_2_ | 0.3179 | 10 |  |  |
|  | Al^2^ | 16b | (0.18, 0.125, 0.45) | 12.53 | Al^2^Al^1^_1_Zr^1^_1_Al^2^_2_Zr^1^_3_Al^1^_1_Zr^1^_1_ | 0.3058 | 9 |  |  |
|  | Zr^1^ | 16b | (0.185, 0.052, 0) | 15.42 | Zr^1^Al^2^_2_Al^1^_1_Al^2^_1_Al^1^_1_Al^2^_2_Al^1^_1_Al^2^_1_Zr^1^_4_ | 0.3436 | 13 | Zr^1^-cnetered Zr_5_Al_9_ cluster | [Zr_5_Al_9_](Zr) |
| ZrAl | Al^1^ | 4c | (0, 0.424, 0.25) | 13.34 | Al^1^Al^1^_2_Zr^1^_7_Al^1^_2_ | 0.3353 | 11 |  |  |
|  | Zr^1^ | 4c | (0, 0.166, 0.25) | 13.35 | Zr^1^Al^1^_7_Zr^1^_6_ | 0.3353 | 13 | Zr^1^-centered Zr_7_Al_7_ cluster | [Zr_7_Al_7_] |
| Zr_5_Al_4_ | Al^1^ | 2b | (0, 0, 0) | 12.14 | Al^1^Zr^3^_6_Al^1^_2_Al^2^_6_ | 0.3437 | 14 |  |  |
|  | Al^2^ | 6g | (0.630, 0, 0.25) | 10.85 | Al^2^Zr^3^_5_Zr^4^_4_Al^1^_2_Al^2^_2_ | 0.3635 | 13 |  |  |
|  | Zr^3^ | 6g | (0.29, 0, 0.25) | 13.36 | Zr^3^Al^2^_2_Al^1^_2_Al^2^_3_Zr^4^_4_ | 0.3336 | 11 | Zr^3^-centered Zr_5_Al_7_ cluster | [Zr_5_Al_7_](Zr_15/4_) |
|  | Zr^4^ | 4d | (0.333, 0.667, 0) | 11.63 | Zr^4^Zr^4^_2_Al^2^_6_Zr^3^_6_ | 0.3336 | 14 |  |  |
| Zr_4_Al_3_ | Al^1^ | 3j | (0.333, 0.167, 0) | 11.87 | Al^1^Al^1^_4_Zr^1^_4_Zr^3^_2_Zr^2^_2_ | 0.3118 | 12 |  |  |
|  | Zr^1^ | 2h | (0.333, 0.667, 0.25) | 15.65 | Zr^1^Zr^1^_2_Al^1^_6_Zr^2^_2_Zr^3^_3_Zr^2^_1_ | 0.3414 | 14 | Zr^1^-centered Zr_9_Al_6_ cluster | [Zr_9_Al_6_](Al_3/4_) |
|  | Zr^2^ | 1f | (0.667, 0.333, 0.5) | 12.97 | Zr^2^Al^1^_6_Zr^3^_3_Zr^1^_6_ | 0.3415 | 15 |  |  |
|  | Zr^3^ | 1b | (0, 0, 0.5) | 12.97 | Zr^3^Al^1^_6_Zr^2^_3_Zr^1^_6_ | 0.3414 | 15 |  |  |

Table SIII. continued

| ICs | Sites | Wyck. | (*x, y, z*) | *IFCs* (eV/Å^2^) | Primitive cluster (R_0_-R_1_-R_2_-R_3_) | *r* (nm) | *CN* | Principal cluster | Cluster formula |
| --- | --- | --- | --- | --- | --- | --- | --- | --- | --- |
| Zr_3_Al_2_ | Al^1^ | 8j | (0.125, 0.125, 0.21) | 11.20 | Al^1^Al^1^_1_Zr^2^_1_Zr^3^_3_Al^1^_1_Zr^2^_2_Zr^1^_2_ | 0.3029 | 10 |  |  |
|  | Zr^1^ | 4d | (0, 0.5, 0.25) | 6.29 | Zr^1^Al^1^_4_Zr^3^_4_Zr^2^_4_Zr^1^_2_ | 0.3499 | 14 |  |  |
|  | Zr^2^ | 4f | (0.34, 0.34, 0) | 16.73 | Zr^2^Al^1^_6_Zr^1^_4_Zr^2^_1_Zr^3^_4_ | 0.3669 | 15 |  |  |
|  | Zr^3^ | 4g | (0.2, 0.8, 0) | 16.77 | Zr^3^Al^1^_6_Zr^1^_4_Zr^2^_4_ | 0.3669 | 14 | Zr^3^-centered Zr_9_Al_6_ cluster | [Zr_9_Al_6_] |
| P6_3_/mmc-Zr_5_Al_3_ | Al^1^ | 6g | (0.59, 0, 0.25) | 11.53 | Al^1^Zr^1^_4_Zr^2^_3_Al^1^_2_Zr^2^_2_ | 0.3209 | 11 |  |  |
|  | Zr^1^ | 4d | (0.333, 0.667, 0) | 12.59 | Zr^1^Zr^1^_2_Al^1^_6_Zr^2^_6_ | 0.3535 | 14 | Zr^1^-centered Zr_9_Al_6_ cluster | [Zr_9_Al_6_](Zr) |
|  | Zr^2^ | 6g | (0.23, 0, 0.25) | 11.37 | Zr^2^Al^1^_5_Zr^2^_6_Zr^1^_4_ | 0.3535 | 15 |  |  |
| I4/mcm-Zr_5_Al_3_ | Al^1^ | 4a | (0, 0, 0.25) | 14.13 | Al^1^Al^1^_2_Zr^2^_8_ | 0.2889 | 10 | Al^1^-centered Al_3_Zr_8_ cluster | [Al_3_Zr_8_](Al_9/5_) |
|  | Al^2^ | 8h | (0.16, 0.66, 0) | 10.76 | Al^2^Zr^1^_2_Zr^2^_8_ | 0.3152 | 10 |  |  |
|  | Zr^1^ | 4b | (0, 0.5, 0.25) | 12.83 | Zr^1^Zr^1^_2_Al^2^_4_Zr^2^_8_ | 0.3500 | 14 |  |  |
|  | Zr^2^ | 16k | (0.077, 0.218, 0) | 10.72 | Zr^2^Al^1^_2_Al^2^_4_Zr^2^_5_Zr^1^_2_Zr^2^_2_ | 0.3613 | 15 |  |  |
| Zr_2_Al | Al^1^ | 2c | (0.333, 0.667, 0.25) | 11.25 | Al^1^Zr^2^_5_Zr^1^_6_ | 0.319 | 11 |  |  |
|  | Zr^1^ | 2a | (0, 0, 0) | 7.81 | Zr^1^Zr^1^_2_Al^1^_2_Zr^2^_2s_Al^1^_4_Zr^2^_4_ | 0.319 | 14 |  |  |
|  | Zr^2^ | 2d | (0.333, 0.667, 0.75) | 14.71 | Zr^2^Al^1^_5_Zr^1^_6_ | 0.319 | 11 | Zr^2^-centered Zr_7_Al_5_ cluster | [Zr_7_Al_5_](Zr_3_) |
| Zr_3_Al | Al^1^ | 1a | (0, 0, 0) | 9.46 | Al^1^Zr^1^_12_ | 0.3092 | 12 |  |  |
|  | Zr^1^ | 3c | (0, 0.5, 0.5) | 12.25 | Zr^1^Al^1^_4_Zr^1^_8_ | 0.3092 | 12 | Zr^1^-centered Zr_9_Al_4_ cluster | [Zr_9_Al_4_](Zr_3_) |
| TiAl_3_ | Al^1^ | 2b | (0, 0, 0.5) | 12.37 | Al^1^Ti^1^_4_Al^2^_8_ | 0.2884 | 12 |  |  |
|  | Al^2^ | 4d | (0, 0.5, 0.25) | 11.67 | Al^2^Al^2^_4_Al^1^_4_Ti^1^_4_ | 0.2884 | 12 |  |  |
|  | Ti^1^ | 2a | (0, 0, 0) | 18.09 | Ti^1^Al^1^_4_Al^2^_8_ | 0.2884 | 12 | Ti^1^-centered TiAl_12_ cluster | [TiAl_12_](Ti_3_) |
| Ti_2_Al_5_ | Ti^1^ | 1a | (0, 0, 0) | 16.90 | Ti^1^Ti^2^_4_Al^10^_8_ | 0.2849 | 12 | Ti^1^-centered Ti_5_Al_8_ cluster | [Ti_5_Al_8_](Al_9/2_) |
|  | Ti^2^ | 1c | (0.5, 0.5, 0) | 13.17 | Ti^2^Ti^1^_4_Al^10^_8_ | 0.2849 | 12 |  |  |
|  | Ti^3^ | 2g | (0, 0, 0.432) | 16.65 | Ti^3^Al^8^_4_Al^9^_4_Al^12^_4_ | 0.2940 | 12 |  |  |
|  | Al^4^ | 2g | (0, 0, 0.141) | 11.61 | Al^4^Ti^6^_4_Al^10^_4_Al^11^_4_ | 0.2874 | 12 |  |  |
|  | Al^5^ | 2g | (0, 0, 0.285) | 12.00 | Al^5^Ti^7^_4_Al^11^_4_Al^12^_4_ | 0.2864 | 12 |  |  |
|  | Ti^6^ | 2h | (0.5, 0.5, 0.145) | 15.89 | Ti^6^Al^4^_4_Al^11^_4_Al^10^_4_ | 0.2923 | 12 |  |  |
|  | Ti^7^ | 2h | (0.5, 0.5, 0.281) | 15.55 | Ti^7^Al^5^_4_Al^11^_4_Al^12^_4_ | 0.2943 | 12 |  |  |
|  | Al^8^ | 2h | (0.5, 0.5, 0.428) | 12.02 | Al^8^Ti^3^_4_Al^9^_4_Al^12^_4_ | 0.2863 | 12 |  |  |
|  | Al^9^ | 2e | (0, 0.5, 0.5) | 13.35 | Al^9^Al^9^_4_Ti^3^_4_Al^8^_4_ | 0.2859 | 12 |  |  |
|  | Al^10^ | 4i | (0, 0.5, 0.071) | 12.13 | Al^10^Al^10^_4_Al^4^_2_Ti^1^_2_Ti^2^_2_Ti^6^_2_ | 0.2923 | 12 |  |  |
|  | Al^11^ | 4i | (0, 0.5, 0.214) | 13.09 | Al^11^Al^11^_4_Ti^7^_2_Ti^6^_2_Al^5^_2_Al^4^_2_ | 0.2873 | 12 |  |  |
|  | Al^12^ | 4i | (0, 0.5, 0.357) | 10.91 | Al^12^Al^12^_4_Al^8^_2_Al^5^_2_Ti^3^_2_Ti^7^_2_ | 0.2943 | 12 |  |  |
| TiAl_2_ | Al^1^ | 2a | (0, 0, 0) | 13.33 | Al^1^Ti^4^_4_Al^2^_4_Al^3^_4_ | 0.2932 | 12 |  |  |
|  | Al^2^ | 2c | (0.5, 0, 0.5) | 13.43 | Al^2^Ti^4^_4_Al^1^_4_Al^3^_4_ | 0.2907 | 12 |  |  |
|  | Al^3^ | 4h | (0.176, 0, 0.5) | 12.17 | Al^3^Al^3^_2_Ti^4^_4_Al^2^_2_Ti^4^_2_Al^1^_2_ | 0.2932 | 12 |  |  |
|  | Ti^4^ | 4g | (0.351, 0, 0) | 15.17 | Ti^4^Al^1^_2_Al^2^_2_Al^3^_6_Ti^4^_2_ | 0.3144 | 12 | Ti^4^-centered Ti_3_Al_10_ cluster | [Ti_3_Al_10_](Ti_2_) |
| TiAl | Al^1^ | 1d | (0.5, 0.5, 0.5) | 11.40 | Al^1^Ti^1^_8_Al^1^_6_ | 0.4071 | 14 |  |  |
|  | Ti^1^ | 1a | (0, 0, 0) | 12.327 | Ti^1^Al^1^_8_Ti^1^_6_ | 0.4071 | 14 | Ti^1^-centered Ti_7_Al_8_ cluster | [Ti_7_Al_8_](Ti) |
| Ti_3_Al | Al^1^ | 2c | (0.333, 0.667, 0.25) | 13.46 | Al^1^Ti^1^_12_ | 0.289 | 12 | Al^1^-centered AlTi_12_ cluster | [AlTi_12_](Al_3_) |
|  | Ti^1^ | 6h | (0.833, 0.666, 0.25) | 13.03 | Ti^1^Al^1^_2_Ti^1^_6_Al^1^_2_Ti^1^_2_ | 0.2896 | 12 |  |  |

Table SIII. continued

| ICs | Sites | Wyck. | (*x, y, z*) | *IFCs* (eV/Å^2^) | Primitive cluster (R_0_-R_1_-R_2_-R_3_) | *r* (nm) | *CN* | Principal cluster | Cluster formula |
| --- | --- | --- | --- | --- | --- | --- | --- | --- | --- |
| Ti_3_Al_5_ | Al^1^ | 2a | (0, 0, 0) | 11.81 | Al^1^Al^6^_4_Ti^7^_8_ | 0.2786 | 12 |  |  |
|  | Al^2^ | 2d | (0, 0.5, 0) | 13.39 | Al^2^Ti^5^_4_Al^4^_4_Al^6^_4_ | 0.2866 | 12 |  |  |
|  | Al^3^ | 4g | (0.249, 0749, 0) | 12.48 | Al^3^Al^6^_2_Al^4^_2_Al^6^_2_Ti^7^_4_Ti^5^_2_ | 0.2918 | 12 |  |  |
|  | Al^4^ | 4h | (0.377, 0.877, 0.5) | 13.04 | Al^4^Ti^5^_2_Al^2^_2_Al^3^_2_Al^6^_4_Ti^7^_2_ | 0.2897 | 12 |  |  |
|  | Ti^5^ | 4h | (0.117, 0.617, 0.5) | 15.68 | Ti^5^Al^4^_2_Al^2^_2_Al^6^_4_Al^3^_2_Ti^7^_2_ | 0.2962 | 12 | Ti^5^-centered Ti_3_Al_10_ cluster | [Ti_3_Al_10_](Ti_3_) |
|  | Al^6^ | 8i | (0.254, 0.504, 0) | 12.51 | Al^6^Al^3^_1_Al^1^_1_Ti^7^_4_Ti^5^_2_Al^2^_1_Al^3^_1_Al^4^_2_ | 0.2882 | 12 |  |  |
|  | Ti^7^ | 8j | (0.120, 0.120, 0.5) | 15.26 | Ti^7^Ti^7^_2_Al^1^_2_Al^6^_4_Al^3^_2_Al^4^_1_Ti^5^_1_ | 0.2962 | 12 |  |  |
| TiCu_3_ | Ti^1^ | 2a | (0, 0, 0.655) | 13.97 | Ti^1^Cu^2^_4_Cu^3^_8_ | 0.2670 | 12 | Ti^1^-centered TiCu_12_ cluster | [TiCu_12_](Ti_3_) |
|  | Cu^2^ | 2b | (0, 0.25, 0.345) | 10.56 | Cu^2^Ti^1^_4_Cu^3^_8_ | 0.2670 | 12 |  |  |
|  | Cu^3^ | 4f | (0.25, 0, 0.155) | 10.59 | Cu^3^Cu^3^_4_Ti^1^_2_Cu^2^_2_Ti^1^_2_Cu^2^_2_ | 0.2670 | 12 |  |  |
| TiCu_2_ | Cu^1^ | 4d | (0, 0.17, 0.56) | 10.69 | Cu^1^Cu^3^_2_Cu^1^_2_Ti^4^_2_Ti^2^_3_Cu^1^_1_ | 0.2709 | 10 |  |  |
|  | Ti^2^ | 2a | (0, 0, 0.6) | 15.09 | Ti^2^Cu^3^_4_Cu^1^_6_Ti^4^_4_ | 0.3334 | 14 | Ti^2^-centered Ti_5_Cu_10_ cluster | [Ti_5_Cu_10_] |
|  | Cu^3^ | 4e | (0.5, 0.17, 0) | 10.69 | Cu^3^Cu^1^_2_Cu^3^_2_Ti^2^_2_Ti^4^_3_Cu^3^_1_ | 0.2709 | 10 |  |  |
|  | Ti^4^ | 2b | (0.5, 0, 0.5) | 15.08 | Ti^4^Cu^1^_4_Cu^3^_6_ | 0.2630 | 10 |  |  |
| Ti_2_Cu_3_ | Cu^1^ | 2a | (0, 0, 0) | 4.67 | Cu^1^Ti^1^_8_Cu^2^_2_ | 0.2792 | 10 |  |  |
|  | Cu^2^ | 4e | (0, 0, 0.2) | 8.02 | Cu^2^Cu^2^_4_Ti^1^_4_Cu^1^_1_Ti^1^_1_ | 0.2792 | 10 |  |  |
|  | Ti^1^ | 4e | (0, 0, 0.4) | 13.84 | Ti^1^Cu^1^_4_Cu^2^_5_Ti^1^_5_ | 0.314 | 14 | Ti^1^-cenetered Ti_6_Cu_9_ cluster | [Ti_6_Cu_9_] |
| TiCu | Cu^1^ | 2c | (0, 0.5, 0.1) | 9.72 | Cu^1^Cu^1^_4_Ti^1^_5_ | 0.2649 | 9 |  |  |
|  | Ti^1^ | 2c | (0, 0.5, 0.65) | 15.43 | Ti^1^Cu^1^_5_Ti^1^_8_Cu^1^_1_ | 0.3238 | 14 | Ti^1^-centered Ti_9_Cu_6_ cluster | [Ti_9_Cu_6_](Cu_3_) |
| Ti_2_Cu | Cu^1^ | 2a | (0, 0, 0) | 6.61 | Cu^1^Ti^1^_8_Cu^1^_4_ | 0.2944 | 12 |  |  |
|  | Ti^1^ | 4e | (0, 0, 0.339) | 14.39 | Ti^1^Cu^1^_4_Ti^1^_8_ | 0.2944 | 12 | Ti^1^-centered Ti_9_Cu_4_ cluster | [Ti_9_Cu_4_](Cu_1/2_) |
| Ti_3_Cu | Cu^1^ | 1a | (0, 0, 0) | 7.32 | Cu^1^Ti^2^_8_Ti^1^_4_ | 0.2940 | 12 |  |  |
|  | Ti^1^ | 1c | (0.5, 0.5, 0) | 3.29 | Ti^1^Ti^2^_8_Cu^1^_4_ | 0.2940 | 12 |  |  |
|  | Ti^2^ | 2e | (0, 0.5, 0.5) | 8.26 | Ti^2^Cu^1^_4_Ti^1^_4_Ti^2^_4_ | 0.2940 | 12 | Ti^2^-centered Ti_9_Cu_4_ cluster | [Ti_9_Cu_4_](Ti_3_) |

**Table SIV.** Valence electrons contribution from the constituent elements in Zr-Cu/Al and Ti-Cu/Al ICs, including the outermost electrons and the valences behaved in common oxides, chlorides and nitrides.

| Elements | Electronic configuration | Outermost electrons | Oxides, chlorides and nitrides | Common valences |
| --- | --- | --- | --- | --- |
| Cu | 3d^10^4s^1^ | 1 | CuO, Cu_2_O, CuCl_2_ | +1, +2 |
| Zr | 4d^2^5s^2^ | 2 | ZrO_2_, ZrN, ZrCl_4_ | +2, +3, +4 |
| Al | 3s^2^3p^1^ | 1 | Al_2_O_3_, AlN, AlCl_3_ | +1, +3 |
| Ti | 3d^2^4s^2^ | 2 | TiO_2_, TiN, TiCl_4_, TiCl_3_ | +2, +3, +4 |

**Table SV.** Zr-Cu/Al and Ti-Cu/Al ICs’ principal cluster with its coordination number (*CN*), cluster formula, total number of atoms per unit cluster formula (*Z*), electron concentration (*e/a*) and valence electrons’ number per unit cluster formula (*N_e/u_*).

| Alloy system | ICs composition | Principal cluster | Cluster formula | *e/a* | *Z* | *N_e/u_* |
| --- | --- | --- | --- | --- | --- | --- |
| Zr-Cu | ZrCu_5_ | *CN*12 Cu_10_Zr_3_ cluster | [Cu_10_Zr_3_](Cu_5_) | 2.33 | 18 | 42 |
|  | Zr_14_Cu_51_ | *CN*15 ZrCu_15_ cluster | [ZrCu_15_](Zr_159/51_) | 2.43 | 19.12 | 46.47 |
|  | Zr_3_Cu_8_ | *CN*12 Cu_8_Zr_5_ cluster | [Cu_8_Zr_5_](Cu_16/3_) | 2.55 | 18.33 | 46.67 |
|  | Zr_7_Cu_10_ | *CN*12 Zr_5_Cu_8_ cluster | [Zr_5_Cu_8_](Cu_2_Zr_2_) | 2.82 | 17 | 48 |
|  | ZrCu | *CN*14 Zr_7_Cu_8_ cluster | [Zr_7_Cu_8_](Zr) | 3 | 16 | 48 |
|  | Zr_8_Cu_5_ | *CN*11 Zr_6_Cu_6_ cluster | [Zr_6_Cu_6_](Zr_18/5_) | 3.23 | 15.6 | 50.4 |
|  | Zr_2_Cu | *CN*12 Zr_9_Cu_4_ cluster | [Zr_9_Cu_4_](Cu_1/2_) | 3.33 | 13.5 | 45 |
| Zr-Al | ZrAl_3_ | *CN*12 ZrAl_12_ cluster | [ZrAl_12_](Zr_3_) | 3 | 16 | 48 |
|  | ZrAl_2_ | *CN*16 Zr_5_Al_12_ cluster | [Zr_5_Al_12_](Zr) | 3 | 18 | 54 |
|  | Zr_2_Al_3_ | *CN*13 Zr_5_Al_9_ cluster | [Zr_5_Al_9_](Zr) | 3 | 15 | 45 |
|  | ZrAl | *CN*13 Zr_7_Al_7_ cluster | [Zr_7_Al_7_] | 3 | 14 | 42 |
|  | Zr_5_Al_4_ | *CN*11 Zr_5_Al_7_ cluster | [Zr_5_Al_7_](Zr_15/4_) | 3 | 15.75 | 47.25 |
|  | Zr_4_Al_3_ | *CN*14 Zr_9_Al_6_ cluster | [Zr_9_Al_6_](Al_3/4_) | 3 | 15.8 | 47.25 |
|  | Zr_3_Al_2_ | *CN*14 Zr_9_Al_6_ cluster | [Zr_9_Al_6_] | 3 | 15 | 45 |
|  | P6_3_/mmc-Zr_5_Al_3_ | *CN*14 Zr_9_Al_6_ cluster | [Zr_9_Al_6_](Zr) | 3 | 16 | 48 |
|  | I4/mcm-Zr_5_Al_3_ | *CN*10 Al_3_Zr_8_ cluster | [Al_3_Zr_8_](Al_9/5_) | 3.63 | 12.8 | 46.4 |
|  | Zr_2_Al | *CN*11 Zr_7_Al_5_ cluster | [Zr_7_Al_5_](Zr_3_) | 3 | 15 | 45 |
|  | Zr_3_Al | *CN*12 Zr_9_Al_4_ cluster | [Zr_9_Al_4_](Zr_3_) | 3 | 16 | 48 |
| Ti-Al | TiAl_3_ | *CN*12 TiAl_12_ cluster | [TiAl_12_](Ti_3_) | 3 | 16 | 48 |
|  | Ti_2_Al_5_ | *CN*12 Ti_5_Al_8_ cluster | [Ti_5_Al_8_](Al_9/2_) | 3 | 17.5 | 52.5 |
|  | TiAl_2_ | *CN*12 Ti_3_Al_10_ cluster | [Ti_3_Al_10_](Ti_2_) | 3 | 15 | 45 |
|  | Ti_3_Al_5_ | *CN*12 Ti_3_Al_10_ cluster | [Ti_3_Al_10_](Ti_3_) | 3 | 16 | 48 |
|  | TiAl | *CN*14 Ti_7_Al_8_ cluster | [Ti_7_Al_8_](Ti) | 3 | 16 | 48 |
|  | Ti_3_Al | *CN*12 AlTi_12_ cluster | [AlTi_12_](Al_3_) | 3 | 16 | 48 |
| Ti-Cu | TiCu_3_ | *CN*12 TiCu_12_ cluster | [TiCu_12_](Ti_3_) | 3 | 16 | 48 |
|  | TiCu_2_ | *CN*14 Ti_5_Cu_10_ cluster | [Ti_5_Cu_10_] | 3 | 15 | 45 |
|  | Ti_2_Cu_3_ | *CN*14 Ti_6_Cu_9_ cluster | [Ti_6_Cu_9_] | 3 | 15 | 45 |
|  | Ti_3_Cu_4_ | *CN*14 Ti_9_Cu_6_ cluster | [Ti_9_Cu_6_](Cu_6_) | 2.29 | 21 | 48 |
|  | TiCu | *CN*14 Ti_9_Cu_6_ cluster | [Ti_9_Cu_6_](Cu_3_) | 3 | 18 | 54 |
|  | Ti_3_Cu | *CN*12 Ti_9_Cu_4_ cluster | [Ti_9_Cu_4_](Ti_3_) | 3 | 16 | 48 |

**Table SVI.** Al-based QCs’ principal cluster with its coordination number (*CN*), cluster formula, total number of atoms per unit cluster formula (*Z*), electron concentration (*e/a*) and valence electrons’ number per unit cluster formula (*N_e/u_*), calculated via formula *N_e/u_*=(*e/a*)×*Z*, with *e/a*=(1.25^3^*π*)/(3*r*_1_^3^×*ρ_a_*) referring from the cluster resonance model^23,24^.

| Alloy system | Principal cluster | Cluster formula | *Z* | *e/a* | *N_e/u_* | QCs composition | Reference |
| --- | --- | --- | --- | --- | --- | --- | --- |
| Al-Pd-TM  (TM=Re, Mn, Ru) | *CN*12 Re_2_Al_11_ cluster | [RePdAl_11_](Re) | 14 | 1.64 | 23.0 | Al_78.6_Pd_7.1_Re_14.3_ | Theor. 28 |
|  |  |  |  |  |  | Al_78_Pd_7.8_Re_14.2_ | Exp. 31 |
|  | *CN*12 Mn_2_Al_11_ cluster | [MnPd(Al_10_Pd)] | 13 | 1.88 | 24.5 | Al_71.4_Pd_14.3_Mn_14.3_ | Theor. 28 |
|  |  |  |  |  |  | Al_70.2_Pd_13.3_Mn_16.5_ | Exp. 32 |
|  | *CN*12 Re_2_Al_11_ cluster | [RePd(Al_9_Pd_2_)] | 13 | 1.64 | 21.3 | Al_69.2_Pd_23.1_Re_7.7_ | Theor. 28 |
|  |  |  |  |  |  | Al_68.6_Pd_23.1_Re_8.3_ | Exp. 32 |
|  | *CN*12 Ru_2_Al_11_ cluster | [Ru_2_(Al_9.5_Pd_1.5_)] | 13 | 1.58 | 20.5 | Al_73.1_Pd_11.5_Ru_15.4_ | Theor. 28 |
|  |  |  |  |  |  | Al_72.5_Pd_13_Ru_14.5_ | Exp. 33 |
|  | *CN*12 Mn_2_Al_11_ cluster | [Mn_2_(Al_9_Pd_2_)] | 13 | 1.77 | 23.0 | Al_69.2_Pd_15.4_Mn_15.4_ | Theor. 28 |
|  |  |  |  |  |  | Al_70_Pd_15_Mn_15_ | Exp. 34 |
|  | *CN*12 Mn_2_Al_11_ cluster | [MnPd(Al_9_Pd_2_)] | 13 | 1.77 | 23.0 | Al_69.2_Pd_23.1_Mn_7.7_ | Theor. 28 |
|  |  |  |  |  |  | Al_68.2_Pd_22.8_Mn_9_ | Exp. 35 |
| Al-Ni-TM  (TM=Co, Rh, Fe) | *CN*12 Co_3_Al_10_ cluster | [CoNi_2_Al_10_](Co) | 14 | 1.69 | 23.7 | Al_71.4_Ni_14.3_Co_14.3_ | Theor. 28 |
|  |  |  |  |  |  | Al_71_Ni_14.5_Co_14.5_ | Exp. 36 |
|  | *CN*12 Ni_3_Al_10_ cluster | [NiRh_2_Al_10_](Ni) | 14 | 1.67 | 23.4 | Al_71.4_Ni_14.3_Rh_14.3_ | Theor. 28 |
|  |  |  |  |  |  | Al_70.5_Ni_15.5_Rh_14_ | Exp. 36 |
|  | *CN*12 Ni_3_Al_10_ cluster | [Ni_2_FeAl_10_](Ni) | 14 | 1.66 | 23.2 | Al_71.4_Ni_21.4_Fe_7.1_ | Theor. 28 |
|  |  |  |  |  |  | Al_71.5_Ni_23.5_Fe_5_ | Exp. 37 |
| Al-Cu-TM  (TM=Co, Ru, Rh, Fe ) | *CN*12 Rh_3_Al_10_ cluster | [RhCu_2_(Al_9_Cu)](Rh) | 14 | 1.58 | 22.2 | Al_64.3_Cu_21.4_Rh_14.3_  Al_62_Cu_23_Rh_15_ | Theor. 28 |
|  |  |  |  |  |  |  | Exp. 38 |
|  | *CN*12 Co_3_Al_10_ cluster | [CoCu_2_(Al_9_Cu)Co] | 14 | 1.68 | 23.5 | Al_64.3_Cu_21.4_Co_14.3_ | Theor. 28 |
|  |  |  |  |  |  | Al_64.3_Cu_21.9_Co_13.8_ | Exp. 39 |
|  | *CN*12 Ru_2_Al_11_ cluster | [Ru_2_(Al_9_Cu_2_)] | 13 | 1.56 | 20.3 | Al_69.2_Cu_15.4_Ru_15.4_ | Theor. 28 |
|  |  |  |  |  |  | Al_68.1_Cu_16.1_Ru_15.8_ | Exp. 40 |
|  | *CN*12 Ru_2_Al_11_ cluster | [Ru_2_(Al_8_Cu_3_)] | 13 | 1.54 | 20.0 | Al_61.5_Cu_23.1_Ru_15.4_ | Theor. 28 |
|  |  |  |  |  |  | Al_61.6_Cu_24.8_Ru_13.6_ | Exp. 40 |
|  | *CN*12 Fe_2_Al_11_ cluster | [Fe_2_(Al_8_Cu_3_)] | 13 | 1.54 | 20.0 | Al_61.5_Cu_23.1_Fe_15.4_ | Theor. 28 |
|  |  |  |  |  |  | Al_62.3_Cu_24.9_Fe_12.8_ | Exp. 41 |

**Table SVII.** Cluster information for typical BMGs in several glass-forming systems, including the principal cluster with its coordination number (*CN*), cluster formula, total number of atoms per unit cluster formula (*Z*), electron concentration (*e/a*) and valence electrons’ number per unit cluster formula (*N_e/u_*), computed via *N_e/u_*=(*e/a*)×*Z*, with *e/a*=(1.25^3^*π*)/(3*r*_1_^3^×*ρ_a_*) referring from the cluster resonance model^23,24^.

| Alloy system | Principal cluster | Cluster formula | *Z* | *e/a* | *N_e/u_* | BMGs composition | Reference |
| --- | --- | --- | --- | --- | --- | --- | --- |
| Cu-Zr/Hf | *CN*12 Cu_8_Zr_5_ cluster | [Cu_8_Zr_5_](Cu) | 14 | 1.69 | 23.7 | Cu_64.3_Zr_35.7_ | Theor. 29 |
|  |  |  |  |  |  | Cu_64_Zr_36_ | Exp. 42 |
|  | *CN*14 Zr_7_Cu_8_ cluster | [Zr_7_Cu_8_](Zr) | 16 | 1.52 | 24.4 | Cu_50_Zr_50_ | Theor. 29 |
|  |  |  |  |  |  | Cu_50_Zr_50_ | Exp. 43 |
|  | *CN*12 Cu_8_Zr_5_ cluster | [Cu_8_Zr_4_Ti](Cu) | 14 | 1.67 | 23.4 | Cu_64.3_Zr_28.6_Ti_7.1_ | Theor. 29 |
|  |  |  |  |  |  | Cu_64_Zr_28.5_Ti_7.5_ | Exp. 43 |
|  | *CN*12 Cu_8_Hf_5_ cluster | [Cu_8_Hf_5_](Cu) | 14 | 1.69 | 23.7 | Cu_64.3_Hf_35.7_ | Theor. 29 |
|  |  |  |  |  |  | Cu_65_Hf_35_ | Exp. 44 |
| Ni-Nb/Ta-(Zr) | *CN*12 Ni_7_Nb_6_ cluster | [Ni_7_Nb_6_](Ni_3_) | 16 | 1.47 | 23.5 | Ni_62.5_Nb_37.5_ | Theor. 29 |
|  |  |  |  |  |  | Ni_62_Nb_38_ | Exp. 45 |
|  | *CN*12 Ni_7_Nb_6_ cluster | [Ni_7_Nb_5_Zr](Ni_3_) | 16 | 1.49 | 23.8 | Ni_62.5_Nb_31.25_Zr_6.25_ | Theor. 29 |
|  |  |  |  |  |  | Ni_62.5_Nb_32.75~30.25_Zr_4.75~7.25_ | Exp. 46, 47 |
|  | *CN*12 Ni_7_Ta_6_ cluster | [Ni_7_Ta_6_](Ni_3_) | 16 | 1.44 | 23.1 | Ni_62.5_Ta_37.5_ | Theor. 29 |
|  |  |  |  |  |  | Ni_62_Ta_38_ | Exp. 48 |
|  | *CN*12 Ta_7_Ni_6_ cluster | [Ta_7_Ni_6_](Ni_3_) | 16 | 1.49 | 23.8 | Ni_56.25_Ta_43.75_ | Theor. 29 |
|  |  |  |  |  |  | Ni_59_Ta_41_ | Exp. 48 |
| Mg-Cu/Ca-(Zn) | *CN*10 Cu_3_Mg_8_ cluster | [Cu_3_Mg_7_Y](Cu) | 12 | 2.04 | 24.5 | Mg_58.3_Cu_33.3_Y_8.3_ | Theor. 29 |
|  |  |  |  |  |  | Mg_58.5_Cu_30.5_Y_11_ | Exp. 52 |
|  | *CN*15 Mg_12_Cu_4_ cluster | [Mg_11_Y_2_Cu_3_](Cu_3_) | 19 | 1.27 | 24.1 | Mg_57.9_Cu_31.6_Y_10.5_ | Theor. 29 |
|  |  |  |  |  |  | Mg_58.5_Cu_30.5_Y_11_ | Exp. 52 |
|  | *CN*12 Mg_7_Ca_6_ cluster | [Mg_3_Cu_4_Ca_6_](Ca) | 14 | 1.73 | 24.3 | Mg_21.4_Ca_50_Cu_28.6_ | Theor. 29 |
|  |  |  |  |  |  | Mg_22.5_Ca_50_Cu_27.5_ | Exp. 54 |
|  | *CN*14 Ca_9_Zn_6_ cluster | [Ca_9_Zn_3_Mg_3_](Ca) | 16 | 1.47 | 23.5 | Mg_18.75_Ca_62.5_Zn_18.75_ | Theor. 29 |
|  |  |  |  |  |  | Mg_17.5_Ca_62.5_Zn_20_ | Exp. 54 |
| Al-La/Ca-(Cu/Ni) | *CN*15 La_9_Al_7_ cluster | [La_9_Al_3_Cu_4_](La_3_) | 19 | 1.31 | 24.8 | Al_15.8_La_63.1_Cu_21.1_ | Theor. 29 |
|  |  |  |  |  |  | Al_15.7_La_62_Cu_22.3_ | Exp. 51 |
|  | *CN*15 La_9_Al_7_ cluster | [La_9_Al_3_Ni_4_](La_3_) | 19 | 1.31 | 24.8 | Al_15.8_La_63.1_Ni_21.1_ | Theor. 29 |
|  |  |  |  |  |  | Al_15.7_La_62_Ni_22.3_ | Exp. 53 |
|  | *CN*14 Ca_9_Al_6_ cluster | [Ca_9_Al_6_](Ca_3_) | 18 | 1.32 | 23.8 | Al_33.3_Ca_66.7_ | Theor. 29 |
|  |  |  |  |  |  | Al_33.3_Ca_66.7_ | Exp. 49 |
| Pd-Si/P-(Cu/Ni) | *CN*13 Pd_11_Si_3_ cluster | [Pd_11_Si_3_](Pd) | 15 | 1.6 | 24 | Pd_80_Si_20_ | Theor. 29 |
|  |  |  |  |  |  | Pd_82.4_Si_17.6_ | Exp. 50 |
|  | *CN*13 Pd_11_Si_3_ cluster | [Pd_11_Si_3_](CuPd_2_) | 17 | 1.46 | 24.8 | Pd_76.5_Si_17.6_Cu_5.9_ | Theor. 29 |
|  |  |  |  |  |  | Pd_77.5_Si_16.5_Cu_6_ | Exp. 51 |
|  | *CN*9 PPd_8_ cluster | [PPd_4_Ni_4_](P) | 10 | 2.33 | 23.3 | Pd_40_P_20_Ni_40_ | Theor. 29 |
|  |  |  |  |  |  | Pd_40_P_20_Ni_40_ | Exp. 55 |
| Zr-Al-Ni/Co | *CN*11 Ni_3_Zr_9_ cluster | [Ni_3_Zr_9_](Al_2_Ni) | 15 | 1.59 | 23.9 | Zr_60_Al_13.3_Ni_26.7_ | Theor. 29 |
|  |  |  |  |  |  | Zr_60_Al_13.3_Ni_26.7_ | Exp. 56 |
|  | *CN*11 Co_3_Zr_9_ cluster | [Co_3_Zr_9_](Al_2_Co) | 15 | 1.59 | 23.9 | Zr_60_Al_13.3_Co_26.7_ | Theor. 29 |
|  |  |  |  |  |  | Zr_60_Al_13.3_Co_26.7_ | Exp. 57 |


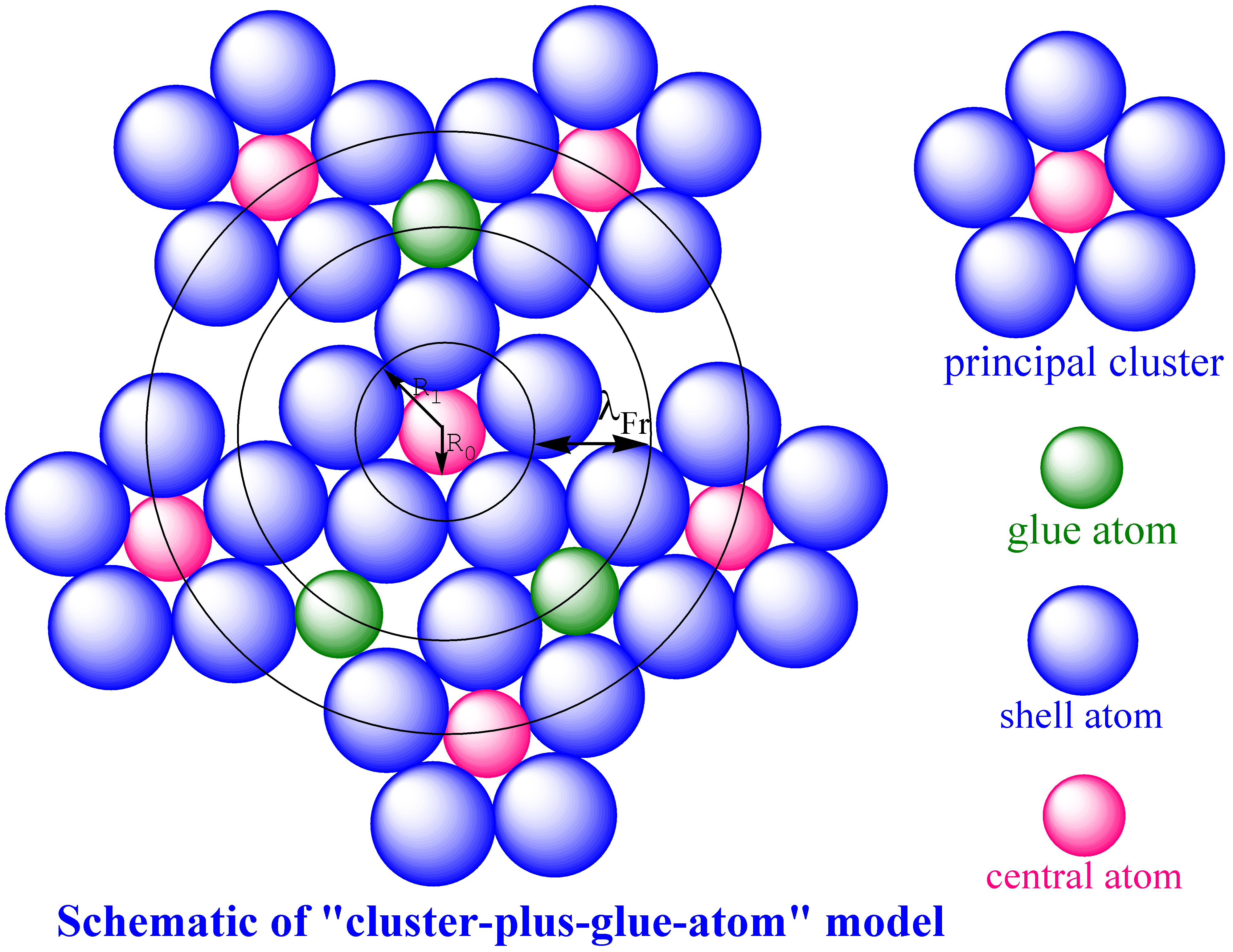


**Figure S1**. Schematic illustration of the “cluster-plus-glue-atom” model, where the atoms are located close to the spherical-shell positions.


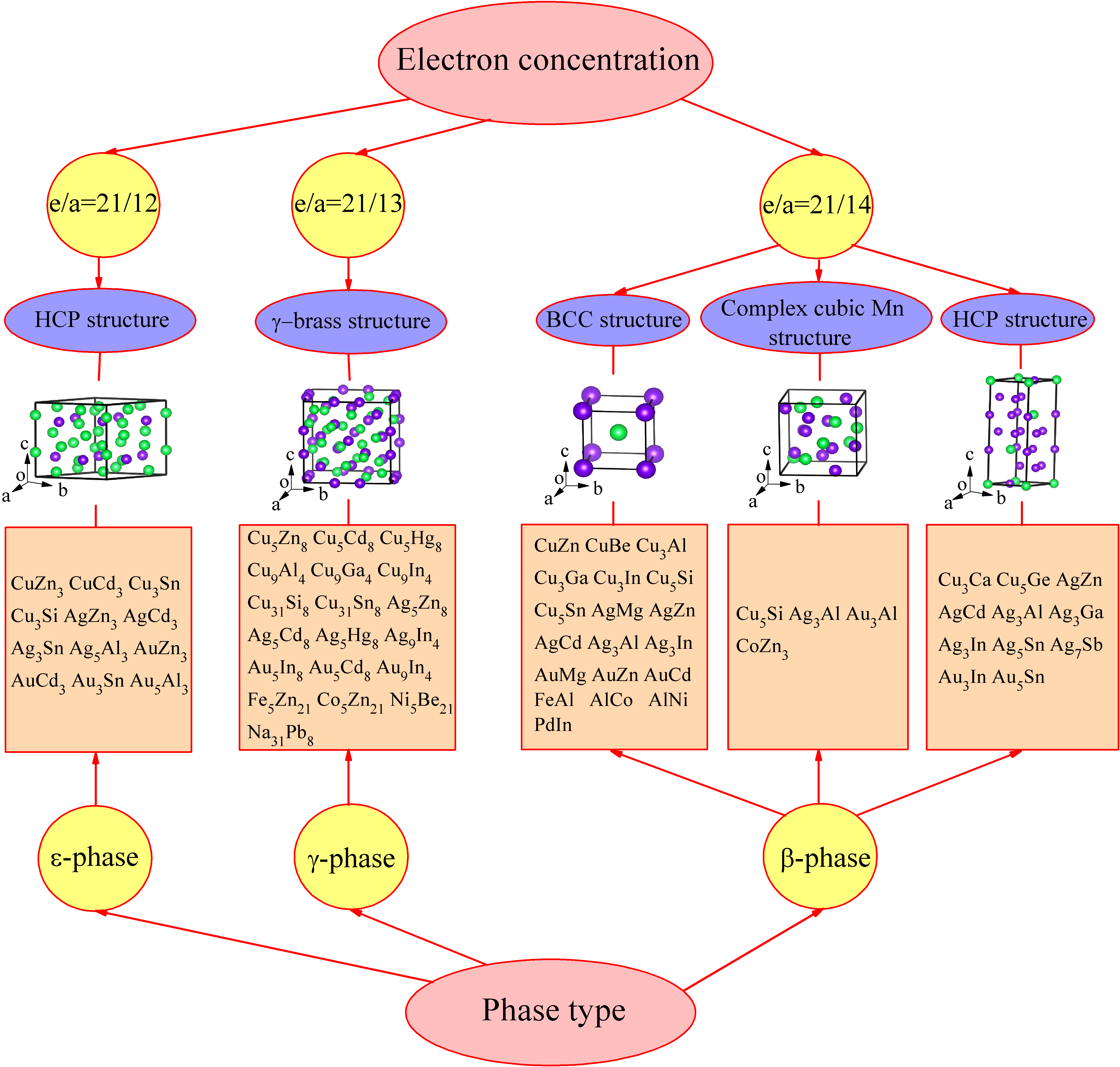


**Figure S2.** Hume-Rothery alloys related electron concentration (*e/a*) and the corresponding structures.

**

**

**
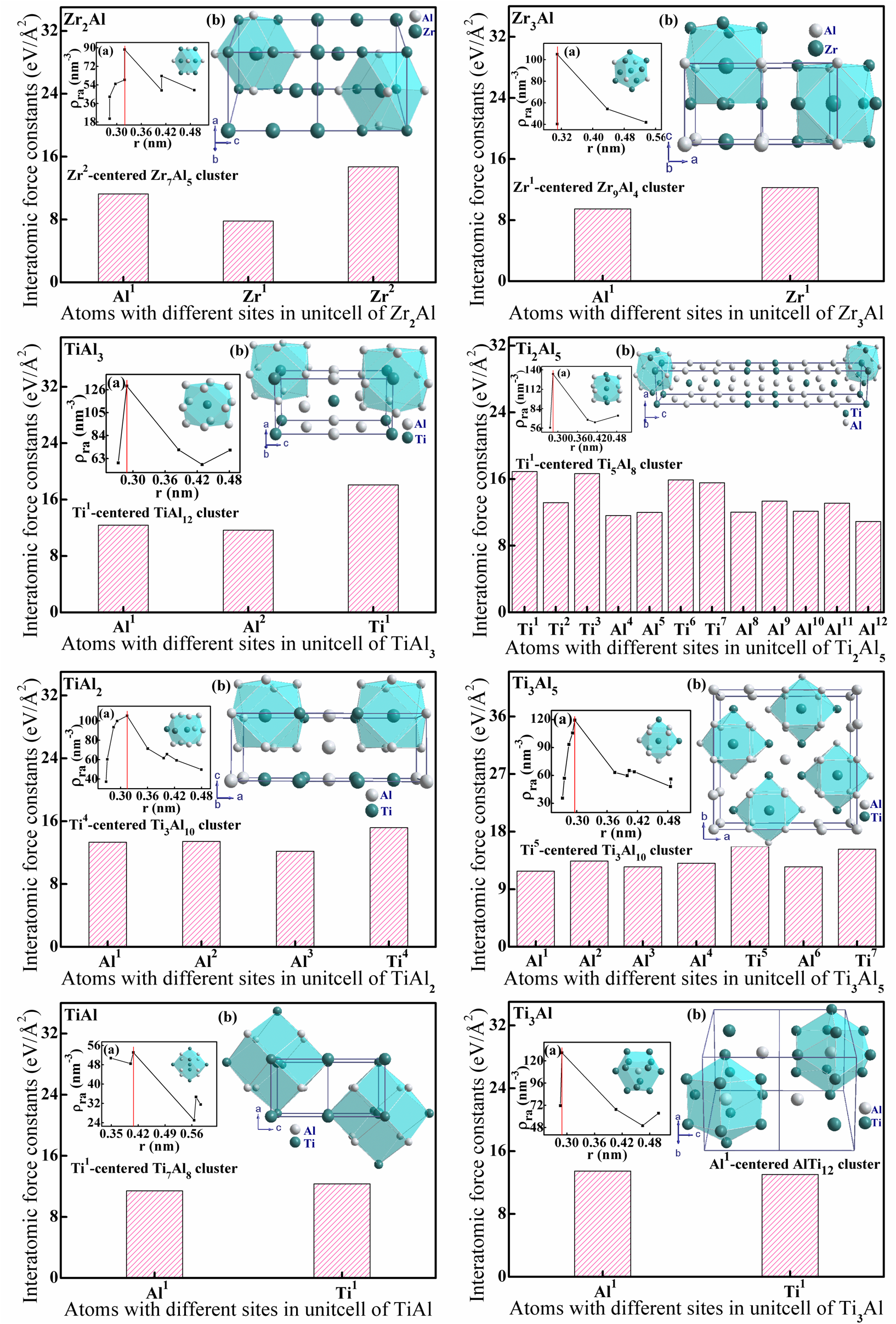
**

**
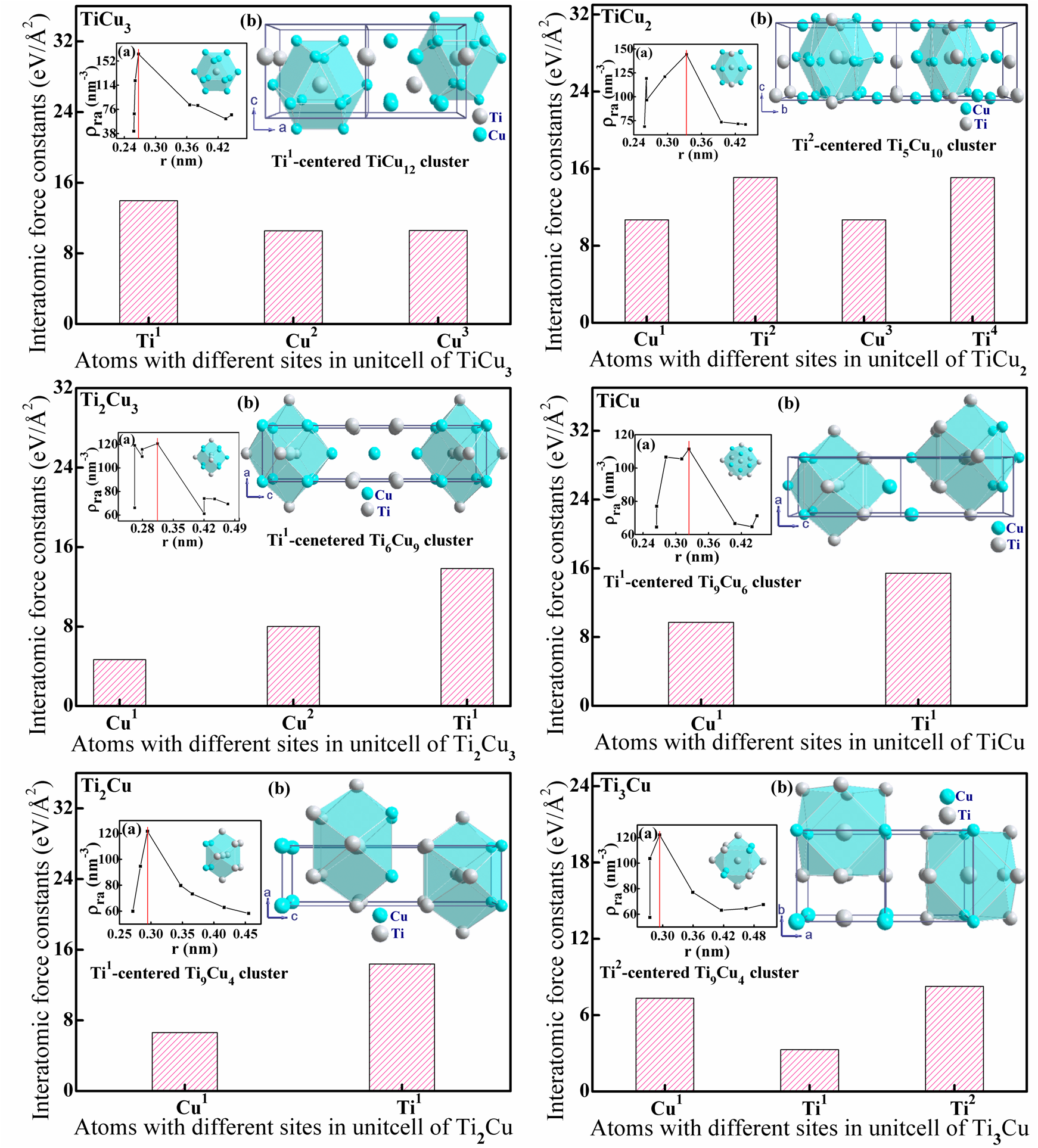
**

**Figure S3**. Principal clusters for the Zr-Al ICs, Ti-Al ICs and Ti-Cu ICs, and their interatomic force constants (*IFCs*). (a) Correlation between radial distances (*r*) and radial atomic density (*ρ_ra_*), the red solid vertical line depicts the cutoff radius of the principal cluster, (b) Atomic clusters present in the crystal structures of these Zr-Al ICs, Ti-Al ICs and Ti-Cu ICs.


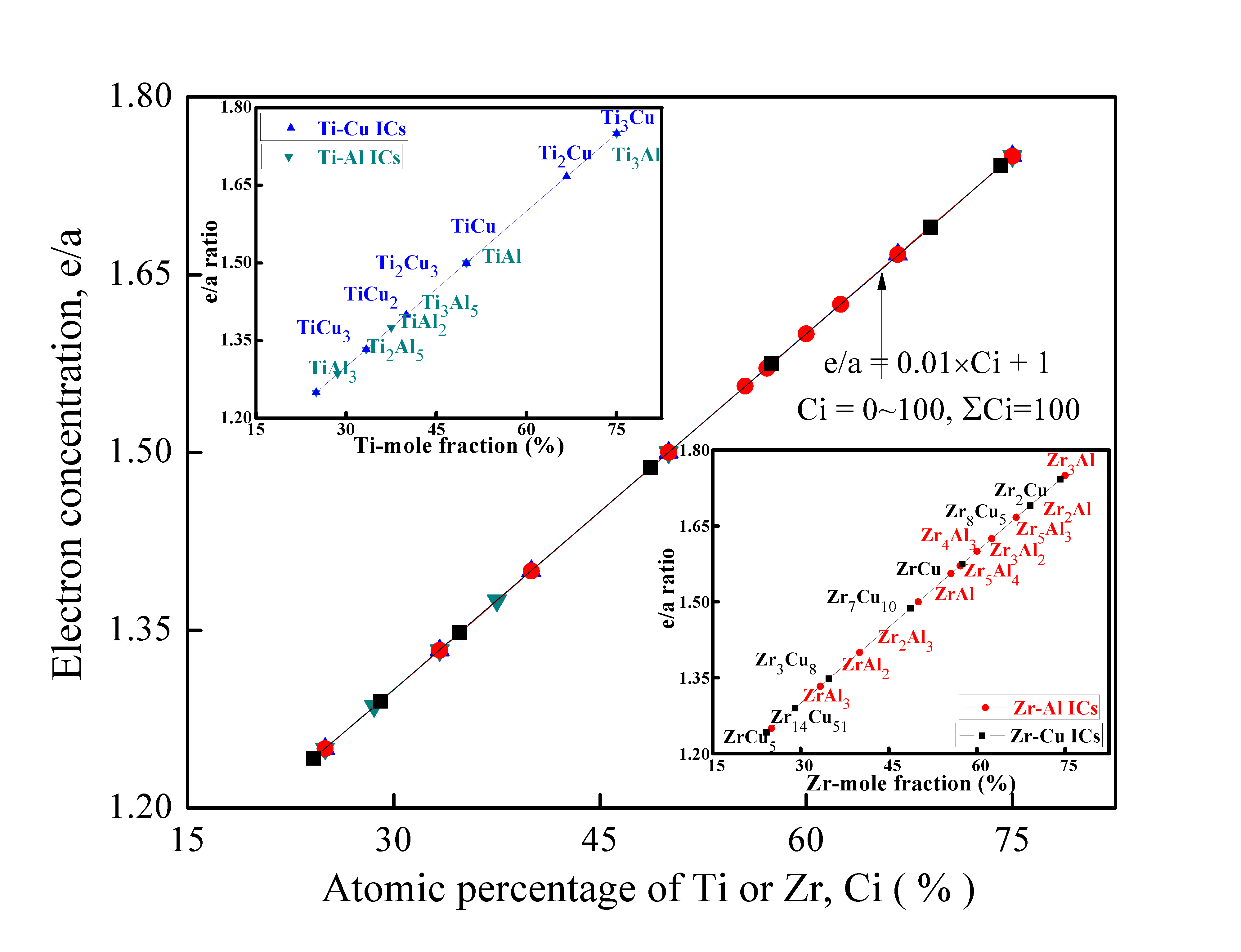


**Figure S4.** Correlation between electron concentration (*e/a*) and Zr/Ti-contents (*C_i_*) for the Zr-Cu/Al ICs and Ti-Cu/Al ICs with apparent cluster features.


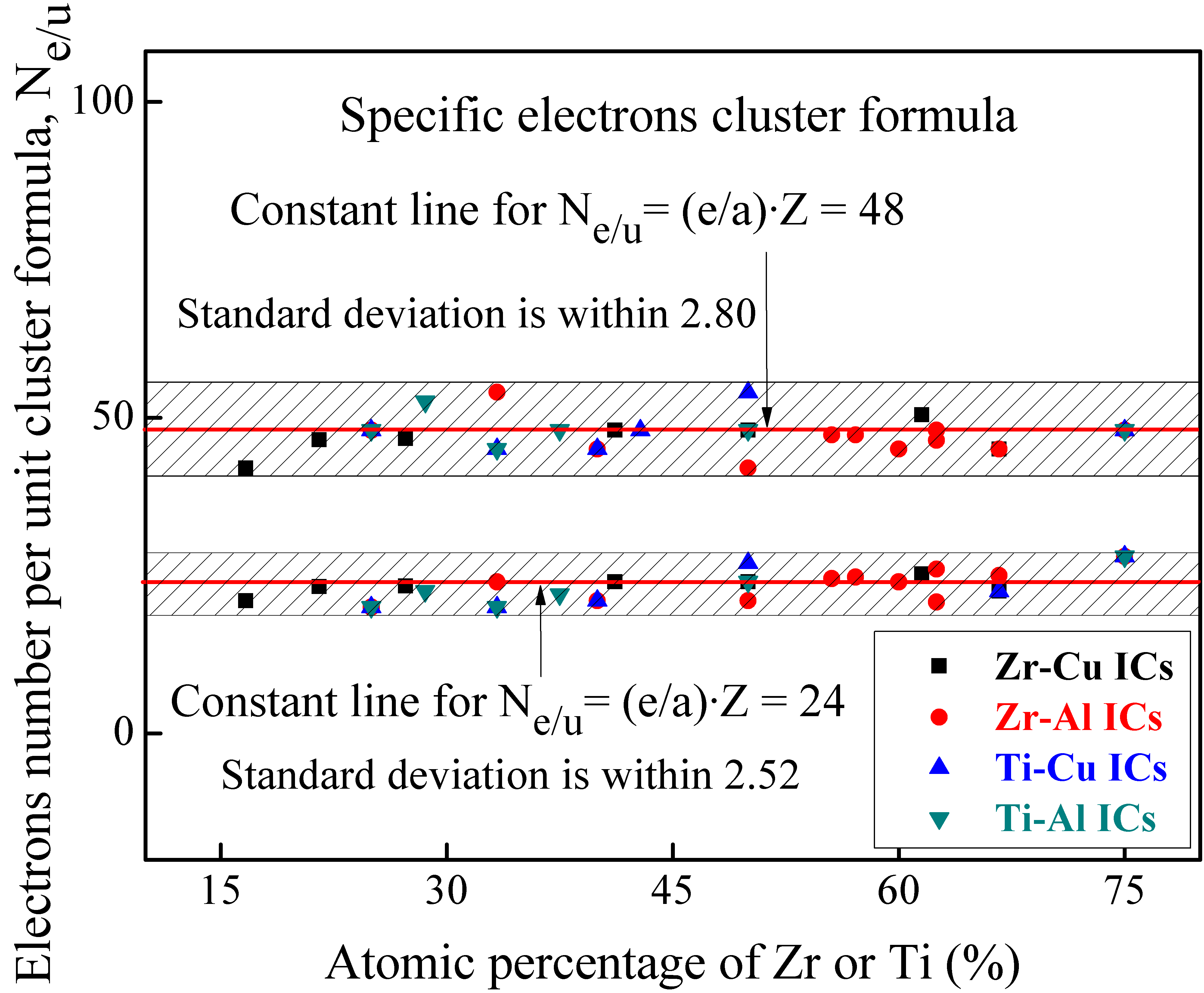


**Figure S5.** Specific electrons cluster formula for the Zr-Cu/Al and Ti-Cu/Al ICs, with the valence electrons’ number per unit cluster formula (*N_e/u_*) close to the specific constants of eight-multiples and twelve-multiples, reflected by the correlations between *N_e/u_* values and Zr/Ti-atomic percentage. It shows the *N_e/u_* values’ deviation from the specific constants of eight-multiples and twelve-multiples 24 and 48, from which the *e/a*-ratio is calculated by adopting the outermost electrons and the common valences as the valence electrons contribution (*e/a*)*_i_* from i element, respectively.


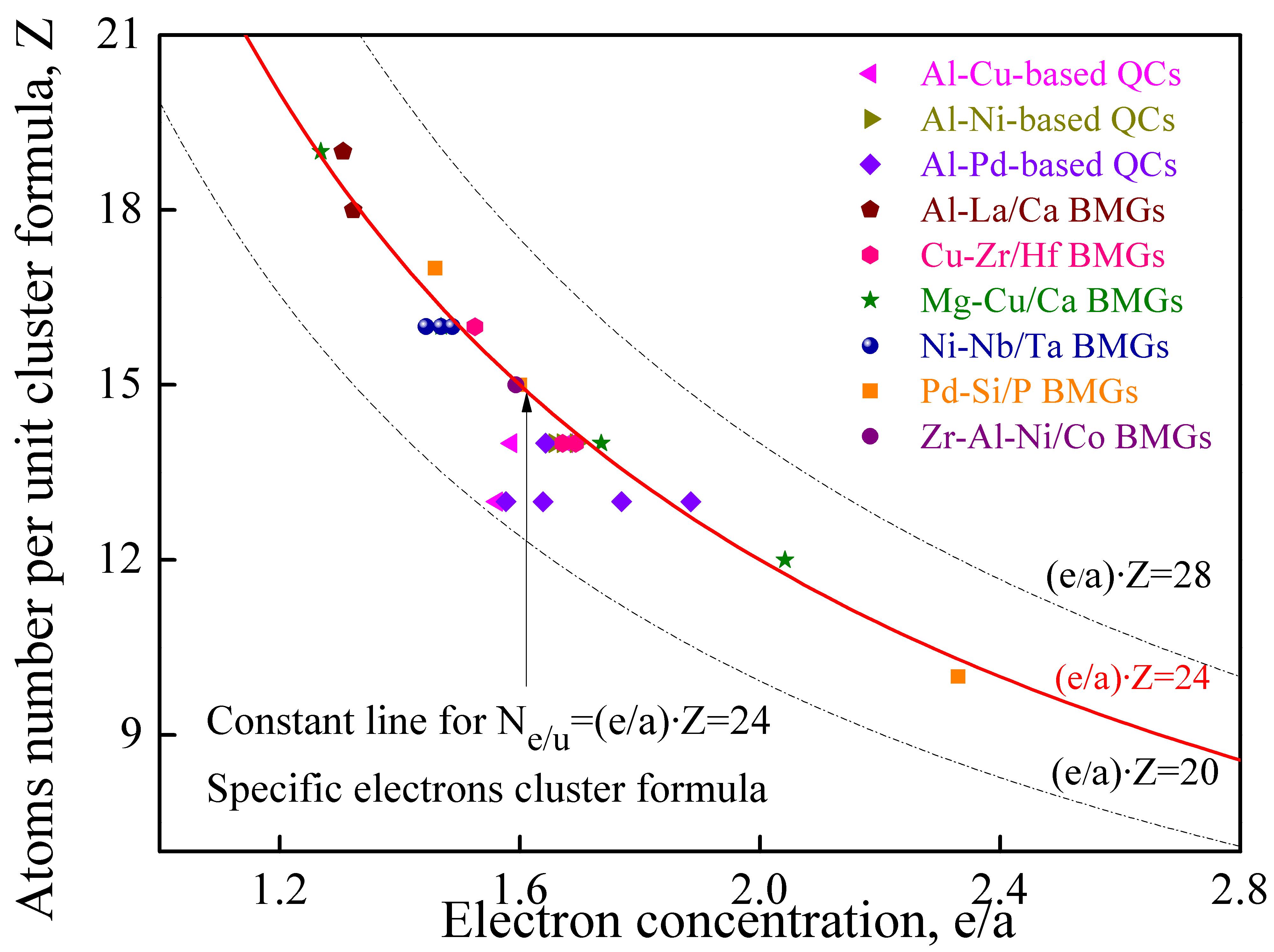


**Figure S6.** Correlation between electron concentration (*e/a*) and the total number of atoms per unit cluster formula (*Z*) for typical Al-based QCs^28^ and BMGs in several glass-forming systems^29^, revealing the CPGAMEC rule of specific electrons cluster formula with *N_e/u_* = (*e/a*)×*Z* ≈ 24.
